# GRP75-faciliated Mitochondria-associated ER Membrane (MAM) Integrity controls Cisplatin-resistance in Ovarian Cancer Patients

**DOI:** 10.7150/ijbs.71571

**Published:** 2022-04-11

**Authors:** Jing Li, Fangzheng Qi, Huishan Su, Chuanshan Zhang, Qing Zhang, Ying Chen, Ping Chen, Linjia Su, Yanan Chen, Yuqi Yang, Zhesheng Chen, Sihe Zhang

**Affiliations:** 1 Department of Cell Biology, School of Medicine, Nankai University, Tianjin, 300071, P. R. China.; 2 Department of Pathology, Third Central Hospital of Tianjin Medical University, 83 Jintang Road, Tianjin, 300170, P. R. China.; 3 Tianjin's Clinical Research Center for Cancer, Tianjin Medical University Cancer Institute and Hospital, Tianjin, P. R. China.; 4 Department of Pharmaceutical Sciences, College of Pharmacy and Health Sciences, St. John's University, Queens, New York, NY, 11439, USA.

**Keywords:** mitochondria-associated ER membrane, glucose-regulated protein, Ca^2+^ fluxes, cisplatin-resistance, ovarian cancer

## Abstract

**Background:** Control of ER-mitochondrial Ca^2+^ fluxes is a critical checkpoint to determine cell fate under stress. The 75-kDa glucose-regulated protein (GRP75) is a key tether protein facilitating mitochondria-associated ER membrane (MAM) formation through the IP3R-GRP75-VDAC1 complex. Although GRP75 contributes to cisplatin (CP)-resistance of ovarian cancer (OC), the underlying mechanisms are not clear.

**Methods:** CP-resistant and -sensitive OC cell lines with GRP75 stable modulation were established. Confocal, PLA, co-IP, and TEM analysis were utilized to detect MAM integrity. Live cell Ca^2+^ imaging, intracellular ATP, ROS, and NAD^+^ assays were utilized to investigate ER-to-mitochondrial Ca^2+^ transfer and mitochondrial bioenergetics. Western blot, flow cytometry, CCK-8, Δψm, and mPTP assays were utilized to examine apoptotic cell death. Bioinformatics, patient's specimens, and immunohistochemistry were conducted to obtain the clinical relevance for GRP75-facilitated MAM formation.

**Results:** GRP75-faciliated MAM formation was enriched in CP-resistant OC cells. CP-exposure only increased MAM formation in CP-sensitive OC cells, and enrichment of GRP75 and VDAC1 at MAMs is indispensable to CP-resistance. Diminishing MAM integrity by GRP75-deficiency reduced ER-to-mitochondria Ca^2+^ transfer, accelerated CP-induced mitochondrial dysfunction, provoked catastrophic ROS, and enhanced CP-triggered apoptotic cell death in OC cells. Clinical investigations confirmed the enrichment of GRP75-faciliated MAM formation in relapsed OC patients, and such enrichment was associated with the CP-resistance phenotype.

**Conclusion:** GRP75-overexpression confers CP-resistance by distinctively managing MAM-facilitated Ca^2+^ fluxes and the pro-survival ROS signal, whereas GRP75-deficiency induces cell death via bioenergetic crisis and apoptotic ROS accumulation in OC cells. Our results show that GRP75-faciliated MAM formation is a potential target to overcome CP-resistance of OC.

## Introduction

Ovarian cancer (OC) is a lethal gynecological malignancy. Most OC patients benefit from initial treatments with cytoreductive surgery followed by cisplatin (CP)-based chemotherapy 1. However, approximately 70% of OC patients relapse and develop drug-resistance after long-term use of CP 2. Multilayer alterations have been found to explain the CP-resistance of OC cells, including increased drug efflux, activation of DNA damage repair machinery, and dysregulation of apoptotic signaling 2, 3. Despite the discovery of these endopathic causes, the mechanisms underlying CP-resistance of OC are not clearly understood.

Cancer cells can trigger a cytoprotective response against CP-toxicity by overexpression (OE) of chaperones for proteostasis 4. The 75-kDa glucose-regulated protein (GRP75/Mortalin) is a chaperone primarily localized in mitochondria, but also found in cell membranes, the endoplasmic reticulum (ER), and the mitochondria-associated ER membrane (MAM) 5, 6. The varied subcellular localizations of GRP75 support its functions in different cellular processes, including mitochondrial import, energy generation, stress response, cell-cycle regulation, membrane transport, and vesicle trafficking 7-10. Previous studies indicated that GRP75 possibly mediates CP-resistance by modulating the anti-apoptotic activity of the aurora-A kinase. One of multiple substrates of this mitotic kinase is p73, a p53-related transcription factor controlling expression of pro-apoptotic genes. GRP75-OE confers CP-resistance to lung carcinoma cells via sequestrating p73 and p53 in the cytoplasm 11, 12. In hypoxic cervical cancer and hepatocellular carcinoma cells, GRP75 exerts a dual anti-apoptotic activity by both restraining the pro-apoptotic function of p53 and supporting the anti-apoptotic function of HIF-1α 13, 14. In OC cells, the contribution of GRP75 to CP-resistance was evidenced by a finding that the knock-down (KD) of GRP75 expression increased CP-induced apoptosis 15. Although these studies support that GRP75 determines the CP-resistant phenotype 4, 15-17, the role of GRP75-mediated pro-survival and anti-apoptosis signaling needs to be further clarified.

MAMs are crucial hubs between cancer prosperity and cell death 18-21. In MAMs, GRP75 links the ER membrane to the mitochondrial outer membrane through facilitating the interaction between the ER-bound Inositol 1,4,5-trisphosphate receptor (IP3R) and the mitochondria-bound voltage-dependent anion channel 1 (VDAC1) 6, 21, 22. This GRP75-faciliated ER-mitochondria contact plays an important role in the regulation of Ca^2+^ homeostasis and the interorganellar transduction of death signals 18-20, 23. In astrocytes and adrenal pheochromocytoma cells, OE of GRP75 after exposure to cellular stress prevents cell death 24, 25. In contrast, GRP75-OE in neuronal cells make them more susceptible to cell death from oxidative glutamate toxicity 26. This suggests that the GRP75-medated MAM formation is important for cell death pathways but present opposite behaviors to stress-induced injury depending on the cell type and cellular state. Contradictory to the facts that GRP75 promotes CP-resistance, GRP75 present in MAMs can facilitate ER-mitochondrial Ca^2+^ fluxes, and excessive Ca^2+^ transferred into mitochondria may cause apoptosis in non-malignant cells and cancer cells sensitive to chemotherapeutic drugs 21, 27. However, altered Ca^2+^ signaling in MAMs is an important hallmark of certain kinds of cancer cells given that it can affect cellular metabolism and resistance to cell death. These cancer cells can gain resistance against cell death by modifying the ER-mitochondria contact 26, 28. Thus, high expression of GRP75 in OC contributes to CP-resistance through the exact mechanism that is a mystery. Although MAM integrity is required for insulin signaling and is implicated in hepatic insulin resistance 29, 30, no study connecting MAM-integrity with CP-resistance of OC was reported.

In this study, we unexpectedly found that GRP75-faciliated MAM formation was enriched in CP-resistant OC cells. CP-exposure enriched GRP75-faciliated MAM formation in CP-sensitive OC cells, and such enrichment was up-regulated in relapsed OC patients and correlated with the CP-resistance phenotype. Further deficiency of GRP75 impaired MAM integrity, reduced ER-to-mitochondria Ca^2+^ transfer, accelerated CP-induced mitochondrial dysfunction, and enhanced the CP-triggered apoptosis in OC cells.

## Materials and methods

### Cell cultures, antibodies and chemicals

CP-sensitive OC cell lines (SKOV3 and A2780) were obtained from the Type Culture Collection of the Chinese Academy of Sciences (China). CP-resistant counterparts (SKOV3/CP and A2780/CP) were obtained from Prof. Ji Zuo and Prof. Liankun Sun 31 15. SKOV3 and SKOV3/CP cells were maintained in RPMI-1640 medium, and A2780 and A2780/CP cells were maintained in DMEM medium, both supplemented with 10% (v/v) fetal calf serum and routinely cultured.

Mouse anti-GRP75 (sc133137) and anti-VDAC1 (sc390996) were obtained from Santa Cruz. Mouse anti-Tubulin (AT819-1) was obtained from Beyolife. Rabbit anti-IP3R (ab108517) was obtained from Abcam. Rabbit anti-Calnexin (CNX) (WL03062), anti-Caspase-3 (WL02117), anti-Caspase-9 (WL03421), anti-Cyto C (WL02410), and anti-Calpain 1(WL03987) were obtained from Wanleibio. Fluo-4 AM (40704ES50), Rhod-2 AM (40776ES50), and ER-Tracker Green (40763ES20) were obtained from Yeasen. Mito-Tracker Red (M7512) was obtained from Life technologies. Mito-Tracker Green (C1048) and pluronic F-127 (ST501) were obtained from Beyolife. MKT-077 (14397) was obtained from Cayman Chemical. Percoll (P8370) was obtained from Solarbio. Protein A/G PLUS-agarose (sc2003) was obtained from Santa Cruz. The enhanced ATP assay kit (S0027), NAD^+^/NADH (WST-8) kit (S0175), JC-1 kit (C2003), mPTP kit (C2009S), enhanced cell counting kit-8 (CCK-8), and annexin V-FITC apoptosis detection kit (C1062L) were obtained from Beyotime. The Amplite™ ROS (Reactive oxygen species) Green Kit (#22900) and MitoROS™ 580 kit (#22971) were obtained from AAT Bioquest. Probenecid (HY-B0545) was obtained from MCE. The Duolink® *In situ* Red Starter Kit (DUO92101) and the remaining fine-grade chemicals were obtained from Sigma.

### Gene stable KD and OE

The CRISPR/Cas9 lentivirus system (LentiCRISPRv2) was used to produce stable GRP75-KD OC cell lines. Three independent GRP75-targeting sgRNAs were generated as previously described 8. The PLV gene-expression lentivirus system (pLV-EF1α-MCS-IRES-GFP-Bsd) was used to produce stable GRP75-OE OC cell lines. GRP75-OE constructs were generated as previously described 32. After co-transfection into 293T cells according to the manufacturer's instructions, the 24 hr-cultured medium was collected, concentrated, and added to and infect OC cells. Clonal cell lines were isolated by culturing single cells in 96-well plates and were screened by Western blotting with the anti-GRP75 antibody.

### Isolation of MAM fractions from cultured cells and clinical tissues

MAM fractions were isolated following the established protocols (Fig. 2A) 33-35. Briefly, OC cells (~2 × 10^9^) or tissues were homogenized on ice. Nuclei and unbroken cells were twice pelleted by centrifugation at 600g for 5 min. The supernatant was collected and centrifuged at 7000g for 20 min to separate crude mitochondria (Mc) from microsomes and the ER fraction. After two washes, the Mc fraction was suspended in 2 ml of mitochondrial re-suspension buffer (MRB, pH 7.4), layered on top of 30% percoll medium, and centrifuged at 95,000g for 30 min. The MAM fraction was extracted from the percoll gradient and further purified by centrifugation to remove contaminated mitochondria. The pure mitochondria (Mp) fraction was collected from the percoll gradient and centrifuged to obtain the MAM pellet. All fractions were stored in -70 °C until use.

### *In situ* proximity ligation assay (PLA)

PLA assays were done using the Duolink® kit following the manufacturer's instructions. Briefly, OC cells grown on glass slides were fixed, permeabilized, and blocked, then incubated overnight with paired primary antibodies (Table S1). After buffer-washing, paired secondary antibodies conjugated with oligonucleotides (anti-rabbit PLUS and anti-mouse MINUS) were applied. If the distance between two targeted proteins were shorter than 40nm, oligonucleotide-conjugating on anti-rabbit PLUS and anti-mouse MINUS were able to connect under the action of a ligase to form a closed circular DNA. After ligation, the signal was amplified by rolling circle amplification (RCA) under the action of a polymerase to form dot-like signals which could be observed under a fluorescent microscope. PLA signals were quantified using the “Particle Analysis” function of Image J software.

OC tissue samples were fixed in formalin and frozen prior to sectioning. 6 μm thick tissue sections were applied for PLA staining as describing above.

### Co-localization analysis of the ER, mitochondria, and Ca^2+^ probe

Co-localization analysis of the ER and mitochondria was done as previously described 36, 37. OC cells grown on glass slides were loaded with Mito-Tracker Red (0.3 μmol/L) and ER-Tracker Green (1 μmol/L) at 37 °C for 30 min. Images were captured with a confocal microscope equipped with a 100× oil immersion objective (excitation at 488 and 594 nm, emission at 505-530, and >560 nm respectively). Co-localization of the ER and mitochondria was quantified as Manders' Co-localization coefficient (MCC) using Image J software with a JACoP plugin. Sixty cells were randomly selected for each condition, and auto-thresholds were applied for both channels to select pixels for co-localization analysis. MCCs were calculated in accordance with the fraction of mitochondrial pixels in contact with the ER (with a higher value representing more co-localization).

Co-localization of the Rhod-2 (red fluorescent signal. 4 μmol/L) with Mito-Tracker Green (0.3 μmol/L) in live cells was performed as described above.

### Live cell Ca^2+^ imaging analysis

The subcellular Ca^2+^ concentration was monitored as previously indicated 37. OC cells were first loaded with the cytosolic Ca^2+^ ([Ca^2+^]_i_) probe Fluo-4 AM (4 μmol/L) or mitochondrial Ca^2+^ ([Ca^2+^]_m_) probe Rhod-2 AM (4 μmol/L) at 37 °C for 30 min, then treated with/without CP and washed with HBSS solution. The Fluo-4 (excited at 488nm, emission at 505-530 nm) or Rhod-2 (excited at 543 nm, emission at > 560 nm) fluorescent signals were viewed under a microscope. 200 frames of 512 × 512 pixels were gathered at 0.98 s/frame by bidirectional scanning mode.

### Transmission electron microscopy (TEM) analysis

OC cell or tissue samples were fixed with 2.5% glutaraldehyde in sodium cacodylate buffer (0.1 mol/L, pH 7.2) at 4 °C overnight and post-fixed for 1 h in 1% osmium tetroxide. Samples were stained for 1 h with 1% uranyl acetate in water before dehydration and embedding in TAAB resin. Images were obtained with a Transmission Electron Microscope. The circumference of each mitochondrion and the proportions of mitochondrial surface closely associated with the ER (<40 nm, distance for ER-mitochondria association) were calculated at high resolution (40,000-80,000×) 34, 38. All identified mitochondria were scored. To obtain an estimate of the minimum distance between sites of ER release and mitochondrial Ca^2+^ uptake, 3 random points from the ER to the nearest mitochondrial outer membrane were drawn with straight lines and measured. Image analysis was performed using Image J software.

### Intracellular ATP, ROS, and NAD^+^ measurement

OC cells were cultured (1x10^4^/well) and treated with/without CP at indicated concentrations. The Intracellular ATP level was checked by the enhanced ATP kit following the manufacturer's instruction. The entire cell population, including any floating cells, was collected, lysated, and boiled to release ATP for the assay. Results were compared to an ATP standard curve with values reported as nmol ATP per mg protein.

Total cytosolic ROS (iROS) levels were determined by the Amplite™ ROS Green Kit following the manufacturer's instruction. Briefly, cells were washed with PBS and incubated with diluted (1:500) Amplite™ ROS Green stock solution at 37 °C in the dark for 60 min. After thorough rinsing with serum-free medium, cells were measured by a fluorescent microplate reader (FLUOstar Omega. Ex/Em = 490/525 nm, cutoff = 515 nm).

Intracellular NAD^+^ levels were measured by using a NAD^+^/NADH kit according to the manufacturer's instruction. Briefly, cold cell lysate was used to determine the total NAD^+^/NADH. To measure the NADH, the cell lysate was incubated at 60 °C for 30 min, alcohol dehydrogenase was added and incubated at 37 °C for 10 min. Then, chromogenic solution was added and incubated at 37 °C for 30 min. A standard curve was generated and assessed for samples. Absorbance values (450 nm) were measured on a microplate reader. The amount of the NAD^+^ was determined by subtracting the NADH from the total NAD^+^/NADH.

### Mitochondrial ROS, ΔΨm, and mPTP measurement

Mitochondrial ROS (mitoROS) level was determined by the MitoROS™ 580 kit with diluted (1:400) MitoROS™ 580 stock solution (Ex/Em=540/590nm, cutoff=570 m). Values compared with the control were regarded as changes in mitoROS levels. Data were recorded from 1 × 10^4^ cells in triplicate per condition.

The mitochondrial membrane potential (Δψm) was determined by the enhanced JC-1 kit following the manufacturer's instruction. Briefly, suspended cells were incubated with an equal volume of JC-1 staining solution (1:400) for 30 min at 37 °C, and then rinsed twice with JC-1 staining buffer. The fluorescent intensity of both JC-1 monomers (FL1-H) and aggregates (FL2-H) was monitored by flow cytometry. The Δψm for each treatment group was calculated as the ratio of red (aggregates) to green (monomers) fluorescence.

The mitochondrial permeability transition (mPTP) level was determined by the MPTP kit according to the manufacturer's instruction. Suspended cells were incubated with an equal volume of Calcein-AM staining solution (1:400) with/without fluorescent quenching solution (CoCI_2_) for 30 min at 37 °C, and collected for checking by flow cytometry.

### Cell viability and apoptosis assay

Cells were seeded at a density of 1x10^4^/ml in 96-well plates for 24 h, with or without CP treatment, and further incubated with 20 μL enhanced CCK-8 solution for 1 h at 37 °C. Absorbance was monitored by a microplate reader (450nm).

Cell death was assessed by annexin V (early apoptosis) and propidium iodide (late apoptosis) double staining using the annexin V-PI apoptosis detection kit followed by flow cytometry analysis. Data were recorded from 1 × 10^4^ cells in triplicate per condition.

### Western blot and Co-immunoprecipitation

OC tissues or cells were lysed in RIPA buffer with a protease inhibitor cocktail. Samples were quantified by the BCA kit, resolved by SDS-PAGE, blotted with primary antibodies (Table S1) and corresponding HRP-conjugated secondary antibodies.

Co-immunoprecipitation (co-IP) from MAM fractions was conducted using Protein A/G PLUS-Agarose. Isolated MAM pellets were re-suspended in 0.1% NP40 lysis buffer. For pre-clearing, 1 μg control IgG was incubated with 20 μL volumes of beads and incubated with MAM lysate at 4 °C for 30 min. The lysate was centrifuged at 4 °C for 5 min at 1000 g, and the supernatant was incubated with the indicated antibody (Table S1) at 4 °C overnight. After adding 20 μL re-suspended volumes of beads at 4 °C for 30 min, the collected beads were washed and precipitated protein was examined by Western blotting.

### Patient samples and immunohistochemistry (IHC)

Seventy-two OC patient's surgical specimens were gathered from the Third Central Hospital and Cancer Hospital of Tianjin Medical University. In addition, five paired specimens (chemo-sensitive and recurrent (chemo-resistant) OC tissues from the same patients) were also collected from the Cancer Hospital of Tianjin Medical University. The histological types, disease stages, and cancer cell contents in each formalin-fixed paraffin-embedded (FFPE) section were examined by experienced pathologists. IHC staining was performed as previously reported 32, 39. Briefly, after deparaffinization, rehydration, and antigen-retrieval treatment with 10 mM sodium citrate buffer (pH 6.0) at 121 °C for 5 min, slides were treated with hydrogen peroxide to quench endogenous peroxidase, blocked with goat serum, and incubated with primary antibodies (Table S1) overnight at 4 °C. Non-immune mouse or rabbit IgG was used as a negative control. IHC staining was performed with the Envision^TM^ two step system (Dako, USA). Slides were treated with 3, 3-diaminobenzidine and counterstained with haematoxylin. Immuno-positivity was independently assessed and scored by two pathologists, who were blinded to the clinical data.

### Bioinformatics analysis

The Kaplan-Meier Plotter (https://kmplot.com), Oncomine (https://www.oncomine.org) and Human Protein Atlas (http://www.proteinatlas.org/) were respectively employed to analyze the expressions of GRP75, VDAC, and IP3R in OC, the overall survival, and their relationship with prognosis. The selected prediction parameter was OS, which was reflected in the median value, and the best segmentation value was automatically selected.

### Statistical analysis

Confocal microscopy, flow cytometry, PLA, and Western blotting data were taken from three independent experiments. All data were reported as mean ± SD, and analyzed using GraphPad Prism 6.0 software. Student's t tests were performed for comparing the two groups of data.

## Results

### GRP75-faciliated MAM formation correlates with CP-resistance of OC cells

**Figure 1:**
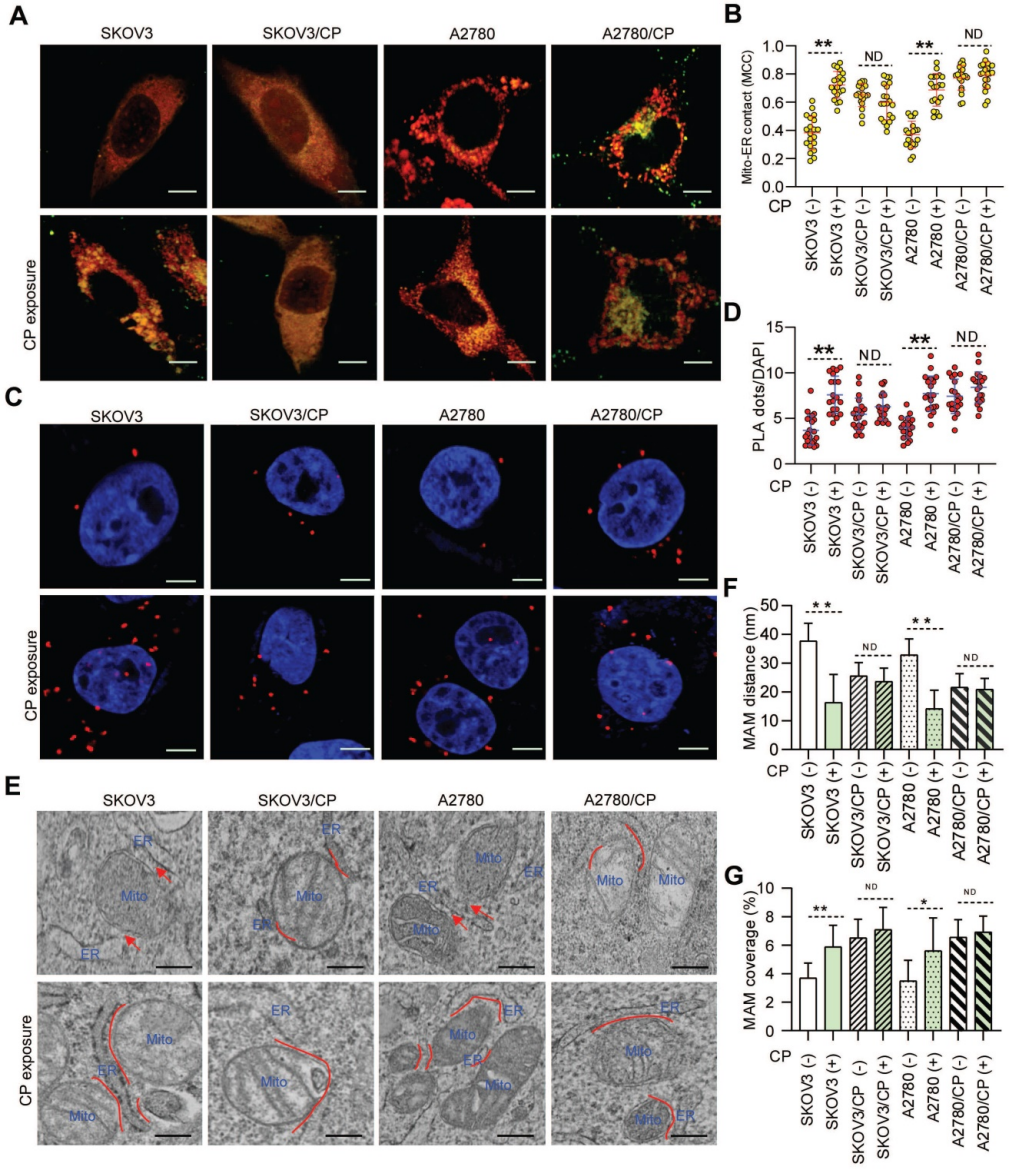
** MAMs were highly presented in CP-resistant OC cells, and CP-exposure increased MAM formation only in CP-sensitive OC cells.** (**A**) Representative confocal images of OC cells stained with Mito-Tracker (red) and ER-Tracker (green). Scale bars: 10 µm; Mito, mitochondria, ER, endoplasmic reticulum. (**B**) Quantification of the MCC of Mito-overlapping with ER. Mean ± SD; n=60 cells per group. Representative images (**C**) and quantification (**D**) of PLA signal (*in situ* close association between IP3R and VDAC1) in OC cells. n=60, Scale bar: 5 μm. (**E**) Representative TEM images of Mito-ER contacts in OC cells. Red lines indicate the contact area of Mito and ER. Red lines with arrow indicate the contact distance of Mito-ER associations. Scale bar: 500nm. Quantification of the mean distance between Mito and ER association (**F**), and the percentage of the Mito-surface close to the ER (**G**) in OC cells (10-20 frames per cell, n=30). Data are mean ± SD from 3 independent experiments, Student t test, **P < 0.01, *P < 0.05.

As ER-mitochondrial tethering at MAMs could impact anti-cancer chemotherapeutic actions 20, 21, 40, we first investigated the correlation between MAM formation and CP-resistance of OC cells. Confocal imaging and PLA assays showed that the MAM number was remarkably higher in CP-resistant OC cells (SKOV3/CP and A2780/CP) than that in CP-sensitive OC cells (SKOV3 and A2780) (Fig. 1A-D). Further TEM checking showed that the intermembrane distance of the ER and mitochondria (Mito) were considerably closer, and the Mito-surface covered with the ER was substantially larger in CP-resistant OC cells (Fig. 1E-G). After CP-exposure on two pairs of cell counterparts (Fig. S1A), a significantly increased MAM number, Mito-ER associated surface, and closer Mito-ER intermembrane distance were observed in CP-sensitive OC cells. No significant changes of the MAM number, Mito-ER distance, and coupling surface were found in CP-resistant OC cells after CP-exposure (Fig. 1). These results suggested that MAMs were highly enriched in CP-resistant OC cells, and CP-exposure increased MAM formation only in CP-sensitive OC cells.

**Figure 2:**
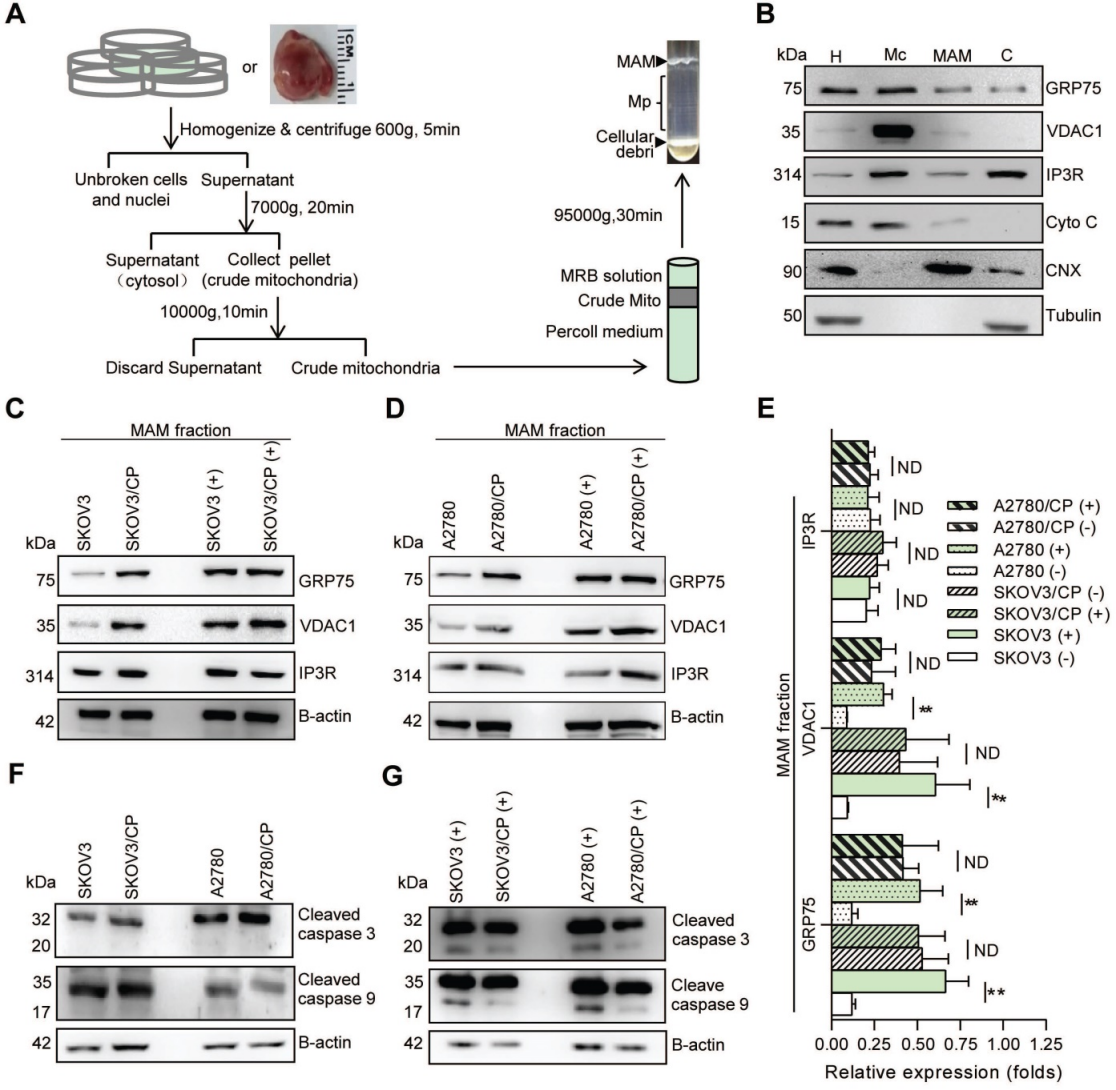
** MAM components were highly enriched in CP-resistant OC cells, and CP-exposure increased the level of MAM components only in CP-sensitive OC cells.** (**A**) Schematic diagram of MAM isolation from OC cells or tissues. MAM were isolated by differential centrifugations and self-forming percoll gradient centrifugations. Other cellular organelles, including crude mitochondria, were also obtained following multiple centrifuge steps. (**B**) Western blot analysis of organelle markers in isolated MAM fractions which were enriched for MAM marker CNX, and free from tubulin and cytochrome C contamination. H: homogenate, Mc: crude mitochondria, C: cytosol. (**C**, **D**) Representative Western blotting of MAM components (GRP75, VDAC1 and IP3R) in isolated MAM fractions of OC cells. (+): CP exposure. 4ug/mL for SKOV3 and SKOV3/CP cells, and 8ug/mL for A2780 and A2780/CP cells. (**E**) Quantification of MAM components in CP-sensitive and -resistant OC cells with/without CP exposure. (**F**, **G**) Western blot analysis of cleaved Caspase in OC cells with/without CP exposure. Data are mean ± SD from 3 independent experiments, Student t test, **P < 0.01.

Because GRP75 facilitates MAM formation through the IP3R-GRP75-VDAC1 complex 6, 22, 26, we next isolated the MAM fraction from OC cells (Fig. 2A,B), and compared the expression levels of MAM components between CP-resistant and -sensitive OC cells. Western blotting results showed that the expression levels of GRP75, VDAC1, and IP3R in whole cellular lysates did not significantly vary among these cells (Fig. S1B-D), whereas the levels of GRP75 and VDAC1 were markedly higher in MAM fractions from CP-resistant OC cells (Fig. 2C-E). When these cell counterparts were exposed to CP, the expression levels of GRP75, VDAC1, and IP3R were slightly reduced in whole cell lysates (Fig. S1B, C). However, CP-exposure significantly increased the levels of GRP75 and VDAC1 in MAM fractions from CP-sensitive OC cells (Fig. 2C-E). No significant changes in GRP75, VDAC1, and IP3R levels were detected in MAM fractions from CP-resistant OC cells after CP exposure (Fig. 2C-E). In parallel, CP exposure only induced the activation of Caspase-3 and -9 in CP-sensitive OC cells, but did not induce their activation in CP-resistant OC cells (Fig. 2F, G). These results suggested that GRP75 was highly enriched in the MAM fraction of CP-resistant OC cells, and CP exposure up-regulated such an enrichment only in CP-sensitive OC cells.

### GRP75-coupled MAM components were highly expressed in CP-resistant OC patients

**Figure 3:**
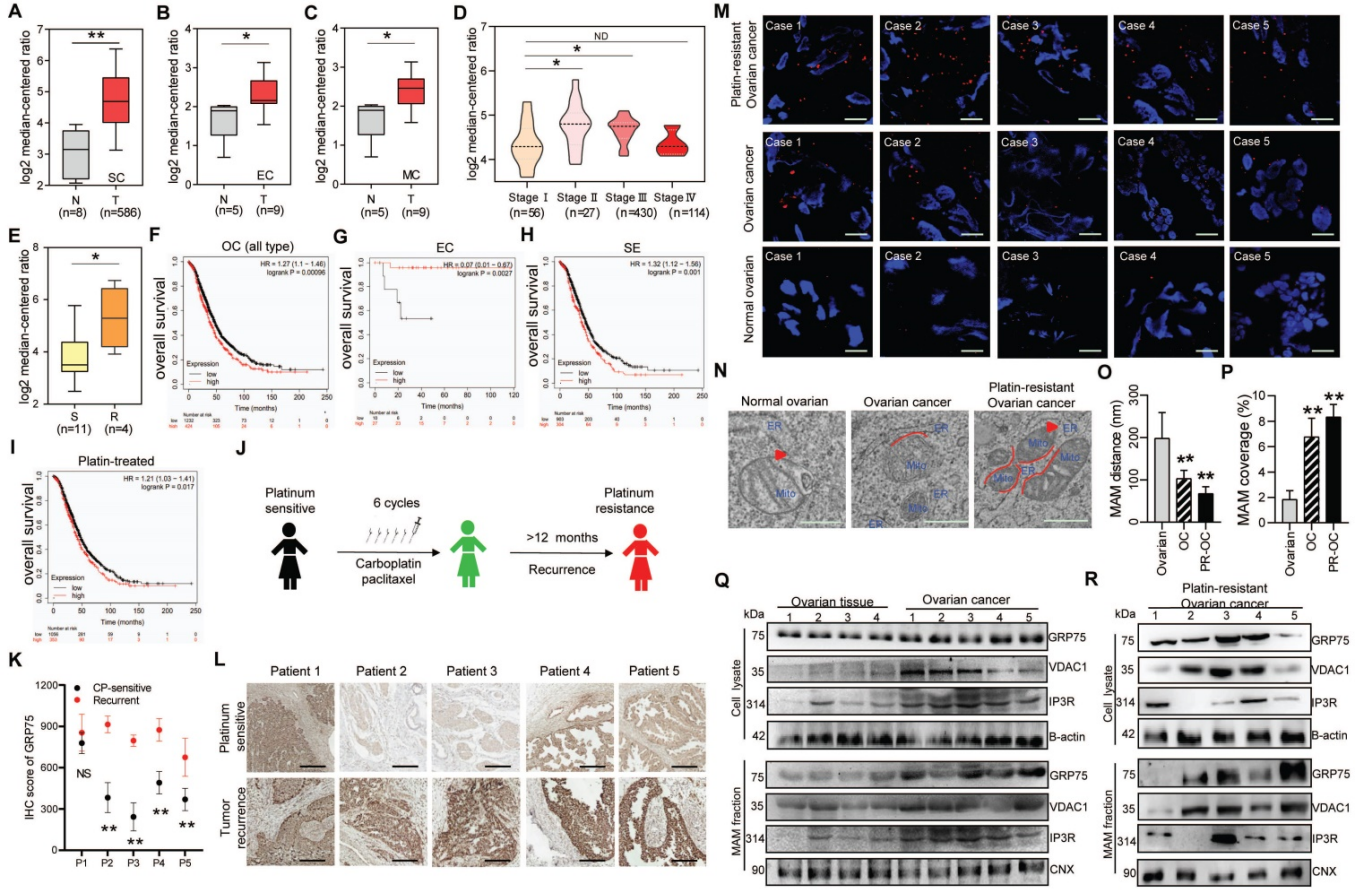
** GRP75-coupled MAM components highly expressed in CP-resistant OC patients.** Oncomine analysis of GRP75 mRNA expression in clinical samples. Box plots depict the expression levels of GRP75 between normal (N) tissues and OC (T) tissues with SC type (**A**), EC type (**B**), MC type (**C**), among different pathological stages (**D**), and between CP-sensitive (S) and -resistant (R) OC cell lines (**E**). Student's t-test, *P < 0.05, **P < 0.01. Survival plots depict the OS for GRP75 low- and high-expressed patients with different OC types: All types (**F**), EC type (**G**), SE type (**H**) and with CP-treatment (**I**). (**J**) Schematic of clinical therapy and monitoring for five OC patients (P1-P5). (**K**) IHC quantification of GRP75 expression in CP-sensitive and recurrent OC tissues (paired samples from the same patient). Data are presented as mean ± SD. ** *P* <0.01. (**L**) Representative IHC images of GRP75 in CP-sensitive (upper panel) and CP-resistant (recurrent, lower panel) OC patients. Scale bar, 100 mm. (**M**) Representative images of PLA signal in tissues of five OC patients before and after treatments. Four frozen-sections of the same tissue sample from different patients were analyzed, n=60, Scale bar: 5 μm. (**N**) Representative TEM images of Mito-ER contacts in tissues of pre-therapeutic and post-treated OC patients. Red lines indicate the contact area of Mito and ER. Red lines with arrow indicate the contact distance of Mito-ER associations. Scale bar: 500 nm. (**O**, **P**) Quantification of the mean distance between Mito and ER association (**O**), and the percentage of the Mito-surface close to the ER (**P**) in OC tissues (10-20 frames per tissue, n=5). Data are mean ± SD, Student t test, **P < 0.01, *P < 0.05. (**Q**, **R**) Representative Western blotting of MAM components in tissue lysate and MAM fraction of pre-therapeutic (**Q**) and post-treated (**R**) OC patients.

Since GRP75-faciliated MAM formation is highly presented in CP-resistant OC cells, we next explored its clinical relevance in OC patients. Oncomine database analysis showed that GRP75 and VDAC1 exhibited higher transcription levels in OC tissues than that in normal ovarian tissues (Fig. 3A-C, Fig. S2A-C). Notably, the transcription of GRP75 and VDAC1, but not IP3R, appeared to be higher in CP-resistant OC cell lines (Fig. 3E, Fig. S2E, N). Further checking of their mRNA levels in different pathological stages of OC showed the transcription of GRP75 and VDAC1, but not IP3R, were significantly up-regulated at advanced stages (II and III) (Fig. 3D, Fig. S2D, M). However, down-regulated transcriptions of GRP75 and VDAC1 were observed at the most advanced pathological stage (IV). To validate these relationships at antigen expression levels, analysis of the OC tissue microarray (from the Human Protein Atlas) was performed. Similarly, GRP75 and VDAC1 were remarkably high-expressed in OC tissues, especially in serous cystadenocarcinoma tissues (Fig. S3A-C). Only a very tiny expression of IP3R was observed in both types of OC tissues (Fig. S3A, D). These sharply contrasted bioinformatics results suggested that main MAM components (GRP75 and VDAC1) were highly expressed in CP-resistant OC patients.

The best way to evaluate the correlation of GRP75-faciliated MAM formation with CP-resistance is to compare the level of MAM components in OC samples from the same patient before CP treatment and after development of CP-resistance. To this end, we identified a total of five OC patients who had received CP-based chemotherapy and had a recurrence due to resistance 12 months post-treatment (Fig. 3J). IHC staining results showed the level of GRP75 was substantially increased in four out of five recurrent OC samples (Fig. 3K, L). Further checking these matched tissues by PLA assays showed that the number of MAMs was significantly increased in most OC tissues, and more markedly increased in recurrent OC tissues (Fig. 3M). TEM analysis showed that the intermembrane distance of Mito-ER was much closer and MAM coupling surfaces were significantly larger in recurrent OC tissues (Fig. 3N-P). To examine the level of change of MAM components from the same OC patient, the MAM fractions from these matched tissues were isolated (Fig. 2A). Western blotting results showed the levels of GRP75, VDAC1, and IP3R from tissue lysates, especially from MAM fractions, were significantly increased in OC tissues (Fig. 3Q, R). Surprisingly, high expressions of GRP75 and VDAC1 were only detected in three cases of OC patients. Noticeably, the three patients (Patient 3, 4 and 5) had been clinically confirmed as CP-resistant OC patients and their IHC analysis indicated that GRP75 levels were markedly increased in resistant OC compared to sensitive OC (Fig. 3L). This case-controlled comparative study suggested that the upregulation of GRP75-facilitated MAM formation is indispensable to CP-resistance of OC.

### High expression of GRP75-coupled MAM components correlates with malignant phenotypes of OC patients

Seeing that high-grade serous ovarian cancer (HGSC) patients frequently relapse with an unfavorable response to CP-based therapy 41, 42, the association of GRP75-coupled MAM component's expression with patient's outcome were further analyzed. The Kaplan-Meier survival prediction showed that a higher mRNA level of GRP75 was significantly associated with shorter OS of the mixed type, the serous cystadenocarcinoma type, and platin-treated OC patients (Fig. 3F, H, I), whereas the mRNA levels of VDAC1 and IP3R were not correlated with the survival of OC patients (Fig. S2F-I, O-R). Strikingly, a negative association of GRP75 transcription with the survival of endometrioid carcinoma patients was found (Fig. 3G).

**Figure 4:**
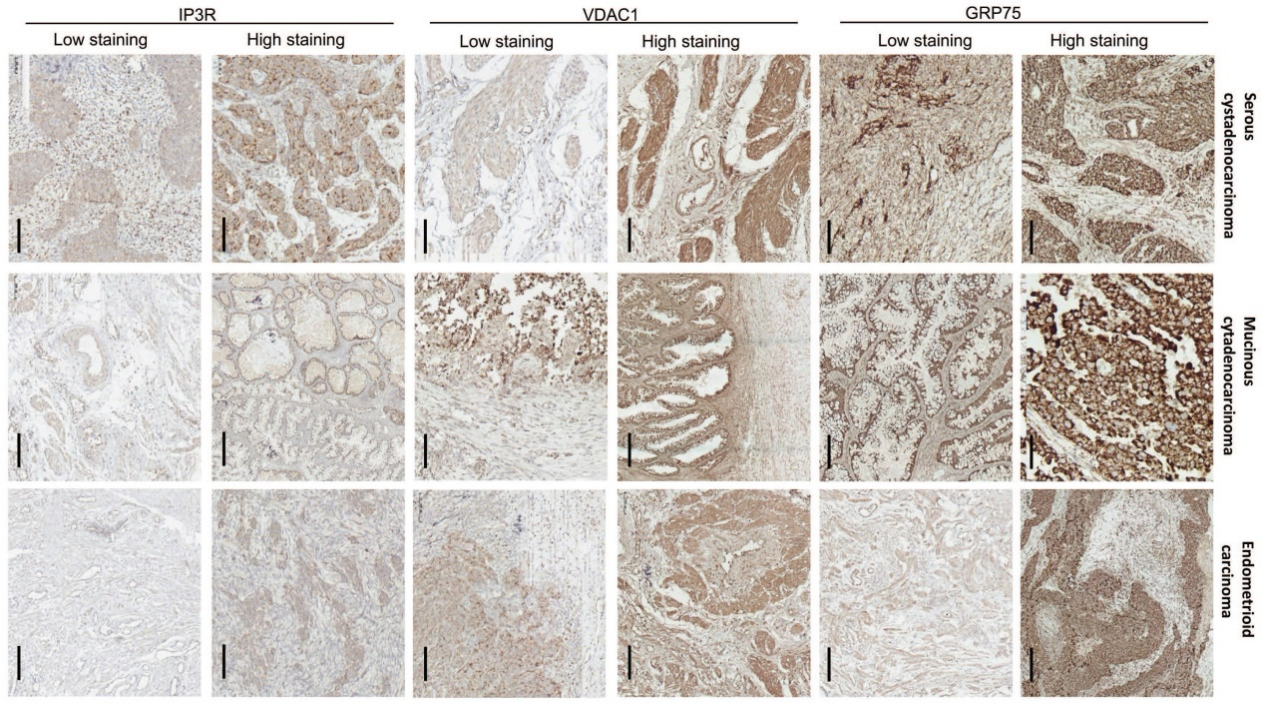
** Expression of GRP75-coupled MAM components in OC clinical samples with different pathological types.** Representative images of varied IHC staining (high- vs low-) of GRP75, VDAC1 and IP3R in OC patients' specimens. Scale bars, 200 µm.

To verify the clinical significance of GRP75-faciliated MAM formation, surgical specimens from OC patients were collected, and the expression profiles of GRP75-coupled MAM components were investigated. IHC staining showed that the expression levels of GRP75, VDAC1, and IP3R varied among OC specimens, each from a different patient. Non-uniform expressions of GRP75, VDAC1, and IP3R were observed in three types of OC specimens, even within the same cancerous lesion (Fig. 4). This may reflect the intra-tumor heterogeneity in OC. The clinicopathological parameters of OC patients at the time of interval debulking surgery are summarized (Table 1). By classifying the specimens based on the expression levels of GRP75-coupled MAM components, we found that low differentiation, small size, metastasis, advanced FIGO and TNM stages of OC were significantly associated with high expressions of GRP75 (Table 1). No association of VDAC1 or IP3R expression with clinicopathological parameters was observed. However, simultaneous high-expression of two or three of GRP75-coupled MAM components exhibited a significantly association with four OC parameters (small size, metastasis, advanced FIGO and TNM stages) (Table S2). Notably, further checking such the associations with routinely examined tumor markers showed that co-expressions, but not individual expression, of GRP75-coupled MAM components were significantly associated with the known CP-resistant marker GST-π (Table 1 and Table S2). These data suggested that a high-expression of GRP75-coupled MAM components may be novel biomarker for predicting CP-resistance in OC patients.

### GRP75 knock-down attenuated MAM integrity and exacerbated CP-induced apoptosis

**Figure 5:**
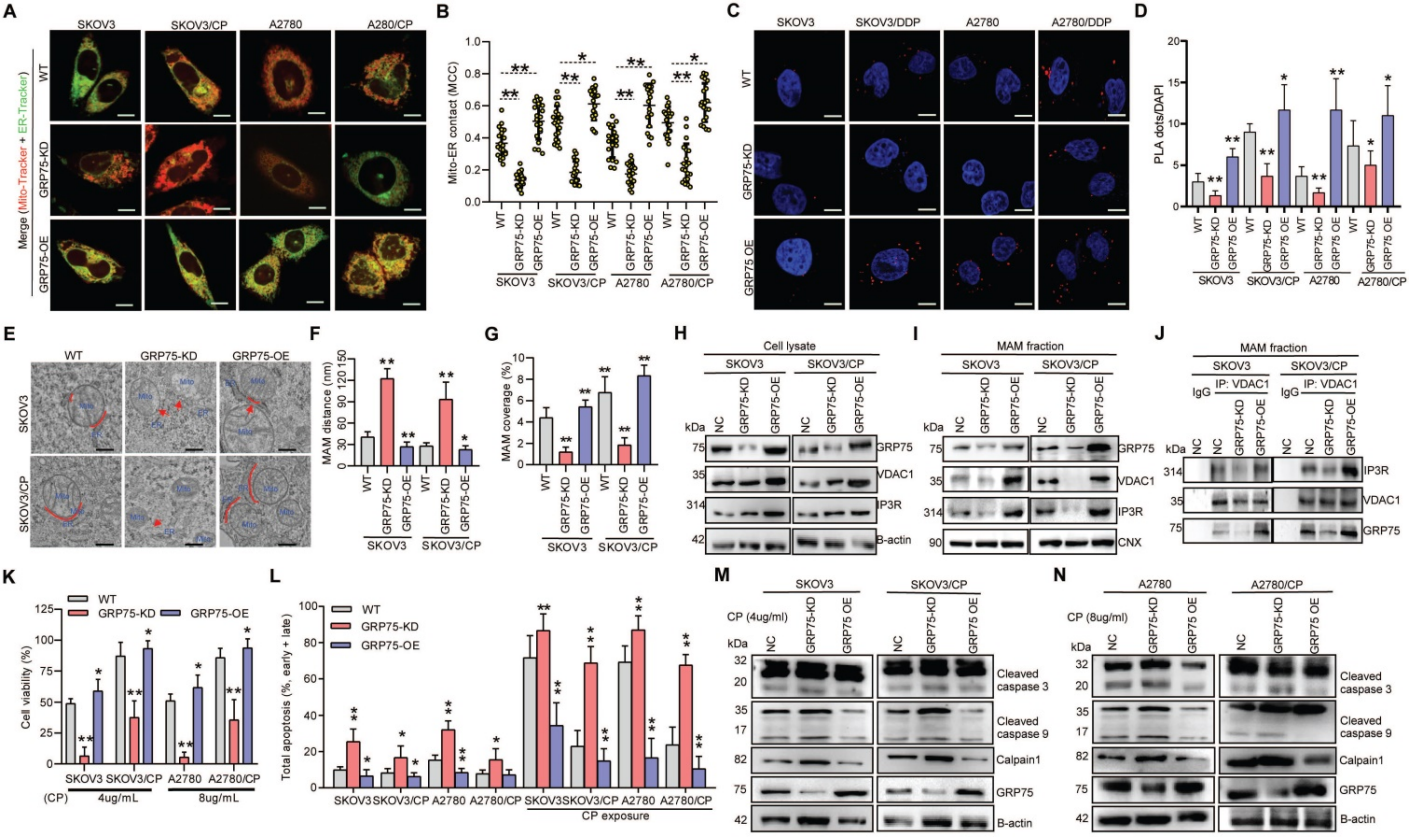
** GRP75-KD weakened MAM coupling degree and exacerbated CP-induced apoptosis in OC cells.** (**A**) Representative confocal images of GRP75-expression-intervened OC cells stained with Mito-Tracker (red) and ER-Tracker (green). Scale bars: 10 μm; (**B**) MCC quantification of Mito-overlapping with ER. Mean ± SD; n=60 cells per group; Representative images (**C**) and quantification (**D**) of PLA signal in GRP75-expression-changed OC cells. n=60, Scale bar: 5 µm; (**E**) Representative TEM images of MAM in GRP75-expression- intervened SKOV3 and SKOV3/CP cells. Red lines indicate the contact area of Mito and ER. Red lines with arrow indicate the contact distance of Mito-ER associations. Scale bar: 500 nm; Quantification of the mean distance between Mito and ER association (**F**) and the percentage of Mito-surface close to the ER (**G**) after the intervention of GRP75 expression (30-50 frames per cell, n=30); (**H**-**J**) Representative Western blotting (**H**, **I**) and co-IP (**J**) of MAM components in GRP75-expression-changed OC cells; (**K**) CCK-8 assay for detecting the cell viability in CP-treated OC cells (CP-sensitive and -resistant) after GRP75 knock-down or overexpression. (**L**) Flow cytometry analysis of the apoptosis level in GRP75-expression-changed OC cells with/without CP exposure. (**M**, **N**) Representative Western blotting of calpain 1, cleaved Caspase in CP-treated (48h) OC cells with GRP75-expression-intervention. Data are mean ± SD from 3 independent experiments. Student t test, **P < 0.01, *P < 0.05, compared to the NC group.

ER-mitochondrial coupling is a key determinant of cell fate in conditions of cellular stress 21, 22, 40. To explore whether GRP75 is critical to maintain MAM integrity and CP-resistance, we first made GRP75-expression-intervened OC cell lines (GRP75-KD and -OE) (Fig. 5), and used these cell lines in the remainder of this study. Following GRP75-KD, the ER-mitochondrial association and MAM number were significantly decreased in CP-resistant and -sensitive OC cells, whereas GRP75-OE markedly increased MAM formation and numbers, especially in CP-sensitive OC cells (Fig. 5A-D). Further TEM checking showed that GRP75-KD markedly enlarged the ER-mitochondrial distance but significantly reduced the Mito-surface covered to the ER in the two pairs of cell counterparts (Fig. 5E-G). Conversely, opposite alterations were found in GRP75-OE cell lines. As GRP75 facilitates MAM formation through the IP3R-GRP75-VDAC1 complex 20, 43, we further checked the GRP75-intervention on protein-protein interaction of MAM components. Western blotting results showed that GRP75-KD or -OE substantially changed the level of VDAC1 and IP3R in MAM fractions, but barely changed their levels in whole cell lysates (Fig. 5H-I, Fig. S4A,B). Furthermore, co-IP results showed that GRP75-KD markedly diminished VDAC1-IP3R interaction, whereas GRP75-OE significantly augmented their interaction in MAM fractions (Fig. 5J, Fig. S4C). These results suggested that GRP75-deficiency impaired the MAM integrity in both kinds of OC cells.

In order to investigate whether impaired MAM formation could affect cell fate, GRP75- intervened OC cell lines were utilized to check the alterations of cell death triggered by CP-exposure. CCK-8 assays showed that GRP75-KD sharply reduced cellular viability in CP-resistant and -sensitive cells, and GRP75-OE significantly increased cellular viability in these cell counterparts (Fig. 5K). FACS-based quantification of CP-exposure-triggered apoptotic cells showed that, in addition to CP-sensitive OC cells, GRP75-KD induced substantial apoptosis in CP-resistant OC cells, and GRP75-OE significantly alleviated CP-induced apoptosis (Fig. 5L). Compared with increased late apoptosis after CP-exposure, GRP75-KD markedly aggravated early apoptosis induced in these cell counterparts (Fig. S5). In parallel experiments, Western blotting results verified that GRP75-KD significantly aggravated CP-induced apoptosis in these cell counterparts, and GRP75-OE significantly inhibited CP-induced cell apoptosis (Fig. 5M-N). These results suggested that GRP75-KD increased the CP-sensitivity of OC cells by increasing apoptosis induction.

### GRP75 knock-down decreased ER-mitochondria Ca^2+^ transfer and accelerated mitochondrial dysfunction

**Figure 6:**
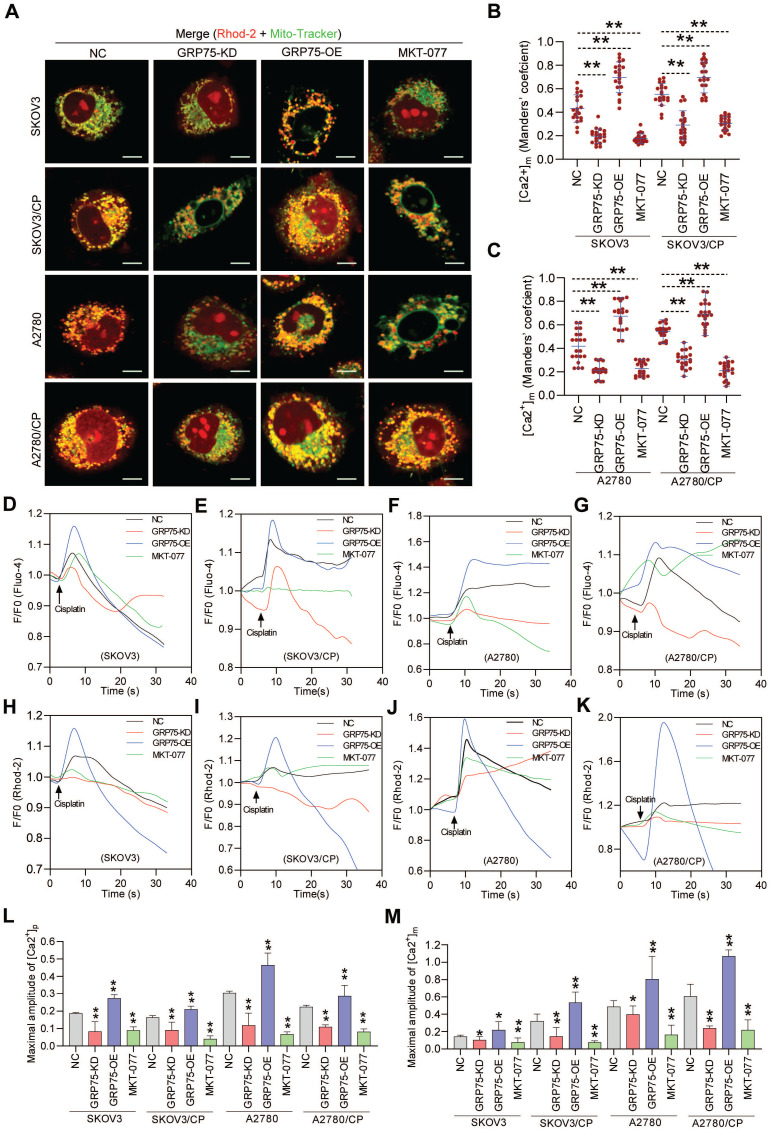
** GRP75-KD or inhibition decreased CP-induced mitochondrial Ca^2+^ uptake.** (**A**) Representative confocal images of Rhod-2 (Red)- and Mito-Tracker (green)-loaded OC cells after GRP75 knock-down, overexpression, or inhibition with MKT-077; (**B**, **C**) Quantification of [Ca^2+^]_m_ level (MCC of Rhod-2 signal overlapping with Mito-Tracker signal) in GRP75-expression-changed, CP-sensitive and -resistant OC cells. Mean ± SD; n=60 cells per group; (**D**-**K**) Representative time-course records of [Ca^2+^]_i_ level (**D**-**G**) and [Ca^2+^]_m_ level (**H**-**K**) in CP-treated OC cells after GRP75-KD, GRP75-OE, or inhibition with MKT-077; (**L**, **M**) Quantifications of the peak amplitude of CP-induced [Ca^2+^]_i_ (**L**) and [Ca^2+^]_m_ (**M**) levels in GRP75-expression-changed, CP-sensitive and -resistant OC cells. Data are Mean ± SD from 3 independent experiments, Student t test, **P < 0.01 compared to the NC group.

Chemotherapeutics-induced cell death depends on the ability to elicit mitochondrial Ca^2+^ overload, and ER-mitochondrial Ca^2+^ fluxes impact cellular sensitivity toward apoptotic stimuli 18, 20, 21, 44. To examine the impact of GRP75-intervention on ER-to-mitochondrial Ca^2+^ transfer, Ca^2+^ imaging assays were conducted (Fig. 6, Fig. S6, S7). Confocal imaging showed that GRP75-KD or inhibition sharply attenuated the [Ca^2+^]_m_ levels in CP-resistant and -sensitive OC cells, whereas GRP75-OE considerably enhanced the [Ca^2+^]_m_ levels in those cells (Fig. 6A-C). Further checking subcellular Ca^2+^ oscillations showed that GRP75-KD or -inhibition significantly attenuated CP-exposure-trigged Ca^2+^ fluxes in mitochondria from these cell counterparts, whereas GRP75-OE substantially increased mitochondrial Ca^2+^ uptake in those cells (Fig. 6H-K, M, Fig. S7). Parallel changes in [Ca^2+^]_i_ oscillations were detected in CP-resistant and -sensitive OC cells after the modulation of GRP75 expression (Fig. 6D-G, L). Remarkably, modulation of GRP75-induced [Ca^2+^]_m_ oscillations changed within wide limits in CP-resistant OC cells (Fig. 6M, Fig. S7). These results suggested that GRP75-KD or inhibition reduced ER-to-mitochondria Ca^2+^ transfer in OC cells.

**Figure 7:**
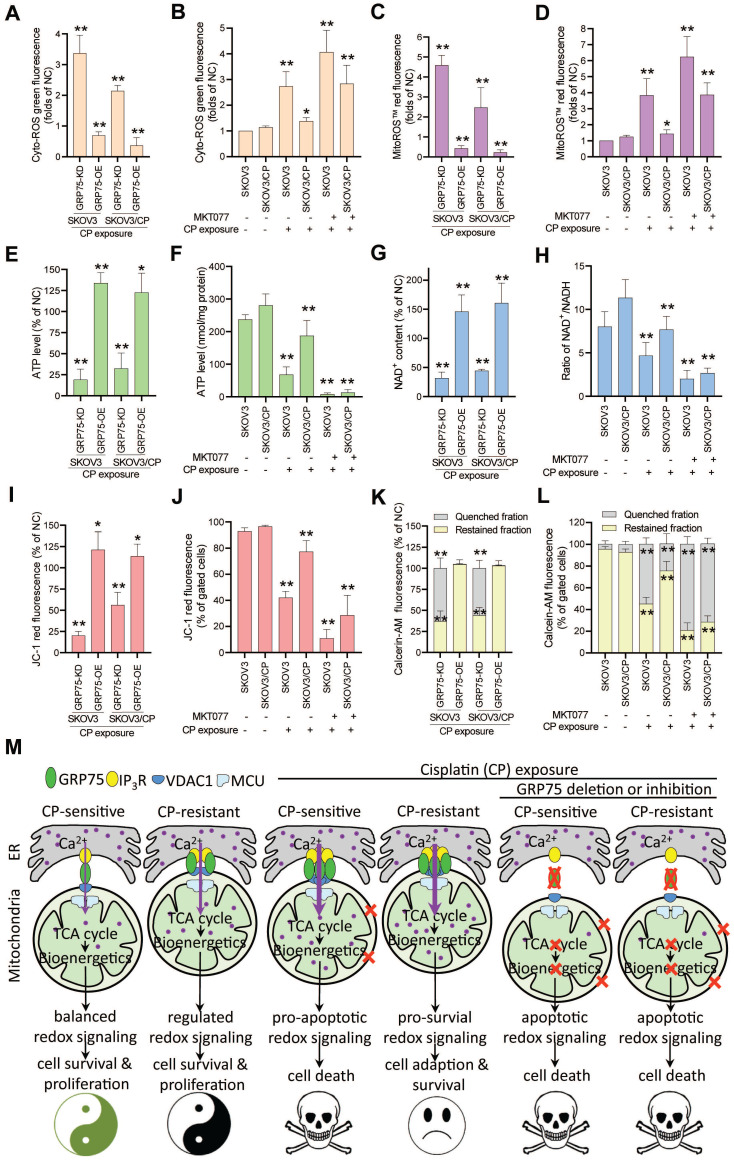
** GRP75-KD or inhibition accelerated CP-induced mitochondrial dysfunction.** (**A**, **C**, **E**, **G**, **I**, **K**) GRP75-expression-changed OC cells were treated with CP (4ug/mL) for 48h. (**B**, **D**, **F**, **H**, **J**. **L**) CP-sensitive and -resistant OC cells were treated with CP (4 µg/mL) and/or MKT077 (40 µM) for 48h. (**A**, **B**) iROS: level was determined by Amplite™ ROS Green staining and measured by a microplate reader. (**C**, **D**) mtROS level was determined by MitoROS™ 580 staining and measured by a microplate reader. (**E**, **F**) The level of intracellular ATP was measured by using the enhanced ATP kit. The ATP level in the NC group of OC cells was set as 100%. (**G**, **H**) Intracellular NAD^+^/NADH ratio was determined by using the NAD^+^/NADH assay kit. The NAD^+^ level in NC group of OC cells was set as 100%. (**I**, **J**) Change in mitochondrial membrane potential was determined by flow cytometry-based measurement of red fluorescence. The ΔΨm in the NC group of OC cells was set as 100%. (**K**, **L**) Quantitative analysis of calcein-AM fluorescent intensity in the presence (quenched) and absence (retained) of CoCl_2_ in OC cells as determined by flow cytometry. The relative calcein fluorescence in the NC group of OC cells was set as 100%. All data represent mean ± SD from three independent experiments. *P < 0.05, **P < 0.01 compared to the NC group or untreated OC cells. (**M**) Working model: GRP75-faciliated MAM integrity represents a checkpoint in regulating the CP-resistance. GRP75-mediated MAM formation boosts ER-mitochondrial Ca^2+^ fluxes, and drives mitochondrial bioenergetics and ROS production, which control the balance of pro-survival and pro-apoptotic signals in OC cells. (**1**) Low-level constitutive ER-to-mitochondria Ca^2+^ fluxes maintain the TCA cycle running, which sustains energy production (ATP), redox homeostasis (NADH generation), anabolic pathways (biosynthesis of macromolecules), and the survival and proliferation of OC cells. (**2**) CP-exposure elicits the damage to nDNA and mtDNA, causes Ca^2+^ overload-release from ER to mitochondria through increased MAM formation, leads to pro-apoptotic mtROS production, mPTP opening and cytochrome C release, triggers activation of Caspases, and induces apoptosis in CP-sensitive OC cells. (**3**) CP-resistant OC cells distinctively manage the [Ca^2+^]_m_ uptake to stimulate Ca^2+^-dependent mitochondrial metabolism and pro-survival mtROS level, while avoiding the Ca^2+^-triggered cell death by fine-tuning GRP75-mediated MAM formation. This probably involves metabolic reprogramming and up-regulated antioxidant enzymes to prevent deleterious [Ca^2+^]_m_ and mtROS accumulation. (**4**) GRP75-deficiency abrogates VDAC1-IP3R1 interaction and ER-mitochondrial coupling, causes ER-to-mitochondria Ca^2+^ transfer-interrupted, mitochondrial dysfunction, compromised OXPHOS (NADH decline), severe bioenergetic crisis (ATP depletion), and eventually results in apoptotic death in CP-sensitive and -resistant OC cells. In these GRP75-depleted cells, CP exposure-induced catastrophic oxidative stress accelerates the mitochondrial dysfunction and cell death.

Control of ER-mitochondrial Ca^2+^ fluxes represents a checkpoint in regulating cell survival and in controlling mitochondrial function 20, 21, 40, 43. To check the action of GRP75-intervention on mitochondrial function, mitochondrial bioenergetics-related processes were determined. ROS measurements showed that GRP75-KD or -inhibition markedly increased iROS and mitoROS levels in CP-treated OC cells (Fig. 7A-D), whereas GRP75-OE significantly decreased iROS and mitoROS levels in those cells (Fig. 7A, C). Analysis of the intracellular ATP level and NAD^+^/NADH ratio found that GRP75-KD or -inhibition significantly reduced ATP and NAD^+^ levels in CP-treated OC cells (Fig. 7E-H), whereas GRP75-OE significantly augmented ATP and NAD^+^ levels in those cells (Fig. 7E, G). Furthermore, FACS-based measurements showed that GRP75-KD or -inhibition markedly lessened Δψm and mPTP levels in CP-treated OC cells (Fig. 7I-L). In contrast, GRP75-OE markedly augmented Δψm and mPTP levels in those cells (Fig. 7I, K). These results suggested that GRP75-KD or inhibition accelerated CP-induced mitochondrial dysfunction in CP-resistant and -sensitive OC cells.

## Discussion

Years of research have established the general view that glucose-regulated proteins (GRPs) overproduced in tumors contribute to malignant phenotypes, including survival, proliferation, metastasis, and multidrug resistance (MDR) 45, 46. However, such a straightforward view is oversimplified, because the expression levels of some GRPs in cancer cells are not upregulated after exposure to CP-included chemotherapeutics 4. Although previous studies directly or indirectly supported the contribution of GRP75 to CP-resistance of OC 15, 47-49, no reports clearly explain how it acted on CP-resistance. Moreover, the role of MAM-resident GRP75 on CP-resistance has not been recognized. For the first time, we present evidence that GRP75-faciliated MAM formation was highly enriched in CP-resistant OC cells. CP-exposure enriched GRP75-faciliated MAM formation only in sensitive OC cells. Furthermore, the MAM integrity was diminished by GRP75-deficiency, which reduced ER-to-mitochondria Ca^2+^ transfer, accelerated CP-induced mitochondrial dysfunction, and promoted CP-triggered apoptotic cell death. Our clinical studies further confirmed the enrichment of GRP75-faciliated MAM formation in relapsed OC patients, and such enrichments were correlated with CP-resistance. These findings revealed that GRP75-mediated ER-mitochondrial coupling determines the CP-resistance of OC patients, and GRP75-targeting-induced MAM disorganization can be used to overcome the CP-resistance of OC cells.

The finding that GRP75-faciliated MAM formation is highly enriched in CP-resistant OC cells and tissues (Fig. 1, 2C-E, 3) is novel and interesting. As CP-exposure to cells increased MAM formation (Fig. 1, 2) and GRP75 present in MAM facilitates ER-mitochondrial Ca^2+^ fluxes, excessive Ca^2+^ transferred into mitochondria might induce apoptosis in CP-resistant OC cells. The reason for apoptosis-resistance favored by the enriched MAM formation in CP-resistant OC cells is not completely understood, but may actually relate to altered Ca^2+^ and ROS signaling. MAM enrichment caused an increase in [Ca^2+^]_m_ (Fig. 6A-C, H-K, M), which might well activate dehydrogenases such as PDH, IDH3, and OGDH, resulting in enhanced NAD^+^ production (Fig. 7G,H) and augmenting oxidative phosphorylation at the electron transport chain (Fig. 7E,F) and finally elevate ROS production (Fig. 7A-D) 50. ROS in cancer cells can exert a double-edged sword role as a pro-survival or pro-apoptosis mechanism, depending on its level and duration of oxidative stress. Theoretically, low concentrations of ROS acting as mitogens induce cell proliferation and survival. Intermediate concentrations of ROS induce cell-cycle arrest, and promote cell differentiation. High concentrations of ROS damage cellular biomolecules, causing mutations, provoke metabolic alterations, adapt stress response, and promote drug-resistance in cancer cells 51. Previous studies indicated activation of the ROS-scavenging signal (e.g., Keap1-Nrf2-ARE pathway) and deregulation of anti-oxidant enzymes (PRDX, GPxs, TrxRs, and MnSOD) in CP-resistant cancer cells 50-52. Because GRP75-OE increased [Ca^2+^]_m_ but decreased mtROS accumulation (Fig. 6H-K, M, Fig. 7C-D), whether GRP75-facilitated MAM enrichment in CP-resistant OC cells is involved in these scavenging/anti-oxidant systems need to be further investigated.

Cancer cells can evolve and gain apoptosis-resistance, by deregulating expression of Ca^2+^-permeable ion channels that rewiring the Ca^2+^-signaling machinery 53, 54, or by modifying ER-mitochondrial Ca^2+^ transfer at MAMs, thereby favoring the MDR phenotype 28, 44, 55. In order to sustain their enhanced proliferation activity, cancer cells require mitochondrial ATP production to meet energy requirements. Since Ca^2+^ oscillation maintains the activity of Krebs cycle dehydrogenases that fuel mitochondrial respiration, proper mitochondrial Ca^2+^ uptake is crucial for cancer cell survival. However, excessive Ca^2+^ uptake in the mitochondria may cause mitochondrial Ca^2+^ overload, which could induce mitochondrial dysfunction and apoptotic cell death. Because balanced ER-mitochondrial Ca^2+^ transfer is controlled by the sophisticated machinery of multiple proteins at the MAMs, cancer cells can gain resistance against cell death by fine-tuning the intermembrane distance and number of MAMs 18, 26, 28. As extra-mitochondrial GRP75 is mostly present in MAMs, its deficiency-induced [Ca^2+^]_m_ declined is primarily due to decreased ER-mitochondrial Ca^2+^ transfer in OC cells (Fig. 6H-K, M). This finding is similar to previous observations in nonmalignant cells (hepatocytes, neurons, and podocytes) that GRP75-silencing attenuates [Ca^2+^]_m_ overload in conditions of oxidative stress 26, 29, 56, 57. However, three distinct points should be clarified: (**1**) CP-induced downregulation of IP3R expression and Ca^2+^-content are closely associated with acquired CP-resistance in cancer cells 58, 59. Here, GRP75-deficiency did not affect the IP3R level in whole lysates of OC cells, but decreased the MAM-resident IP3R level and VDAC1-IP3R interaction (Fig. 5H-J). This implies that GRP75-OE conferred CP-resistance is possible through the IP3R-mediated Ca^2+^ signal from MAMs. (**2**) VDAC1 has an open anion selective state and a closed but slightly cation-selective state. The conductive state of VDAC1 plays a major role in regulating ER-mitochondrial Ca^2+^ transfer and the apoptotic signal 60. In stimulated cells, VDAC1 mediates Ca^2+^ transport from IP3R to the inner mitochondrial membrane and rapid Ca^2+^ uptake by mitochondria 61. GRP75-deficiency decreases the MAM-resident VDAC1 level and VDAC1-IP3R interaction (Fig. 5H-J), supporting that GRP75-deficiency diminishes CP-resistance is possible through attenuating VDAC1-mediated ER-mitochondrial Ca^2+^ transfer and Ca^2+^ uptake into mitochondria. (**3**) Bcl-2 family proteins modulate CP-induced ER-mitochondrial Ca^2+^ transfer by regulating the MAM number 62. Several studies showed that Bcl-2 and Bcl-XL are co-enriched with GRP75 in MAMs 63, 64, and GRP75-deficiency induces their conformation change and Caspase-dependent apoptosis 65-67. Hence, GRP75-deficiency induced apoptotic cell death may be due to the reduced recruitment of Bcl-2 family proteins to MAMs. This possibility is now being checked in our lab.

Mitochondrial Ca^2+^ addiction is vital for the survival and proliferation of cancer cells, which requires constitutive ER-to-mitochondrial Ca^2+^ fluxes boosted by MAM formation 44, 68. Low-level ER-to-mitochondria Ca^2+^ transfer is essential to sustain an adequate tricarboxylicacid (TCA) cycle, which ensures energy production (ATP), redox homeostasis (NADH generation), and anabolic pathways (biosynthesis of fatty acids, amino acids, and nucleotides) 69, 70. Logically, ablation of ER-mitochondrial Ca^2+^ fluxes by GRP75-deficiency results in compromised mitochondrial bioenergetics, causing declines in ATP and NADH levels in both kinds of OC cells (Fig. 7E-H). These elicited effects were comparably toxic to CP-sensitive and -resistant OC cells. Notably, GRP75-deficiency induced a bioenergetic crisis in OC cells, as indicated by increased oxidative stress, depolarized the mitochondrial membrane, and collapsed membrane potential (Fig. 7A-D, I-L). Previous reports revealed that GRP75-KD activates AMPK-dependent, mTOR-independent autophagy, which could enable normal cells to survive 70-72. Although we have not yet checked autophagy variation in the present study, GRP75-deficiency boosted the accumulation of deleterious iROS and mtROS (Fig. 7A-D), and made both kinds of OC cells more susceptible to CP-triggered cell death. Noticeably, the latest studies have identified adenine nucleotide translocase 3 (ANT3) as a novel GRP75 client and can serve as a death effector when GRP75 was depleted in chemo-resistant or -sensitive tumor cells. The upregulated expression and mislocalization of GRP75 in tumors prevent mitochondrial permeability and cell death by inhibiting the interaction between ANT3 and cyclophilin D (CypD) 73, 74. GRP75 depletion increased mitochondrial membrane permeability and selectively induced cancer cell death in BRAF- or KRAS-mutated tumors, which were attenuated by knock-down or inhibition of ANT, CypD, or MCU (mitochondrial Ca^2+^ uniporter) 74, 75. These observations together with our data highlight that GRP75-deficiency induces cell death via severe bioenergetic crisis and apoptotic oxidative stress in OC cells (Fig. 7M).

There are two limitations in the present study: (1) We had no way to do the rescue experiments on CP-resistant OC cells with GRP75-KD or -OE, because those cells were totally dead before performing any readouts. (2) Considering no recognized clinical markers for MAMs, it is a challenge to check the MAM level in tissue samples by routine approaches. However, based on IHC staining information, we found for the first time that co-expression of MAM components (GRP75 and VDAC1) was significantly correlated with malignant characters, in terms of small size, metastasis, advanced FIGO, and TNM stages (Table 1 and Table S2). These data are consistent with previous reports of GRP75 and VDAC1 action on the aggressive phenotypes of OC 8, 47, 76-78. No associated expression of any MAM components with P-gp was found (Table 1 and Table S2). This may be because P-gp expression was vary variable and less responsive to CP-treatment in OC patients 79, 80. Remarkably, co-expression of MAM components associated with the well-known CP-resistant marker GST-π is exciting (Table 1 and Table S2). This may be attributed to CP-resistant OC patients showing high expression of glutathione-S-transferases 81, 82. These clinical data well mirror previous reports that GRP75 and VDAC1 are CP-resistance-associated proteins 47, 48, 83, and fully vindicate their coupling action on promoting CP-resistance. However, further work should pursue specifically targeting GRP75 at MAM to solidify the present finding.

In summary, we define a critical checkpoint role of GRP75-faciliated MAM integrity in determining CP-resistance of OC. Our findings suggest that GRP75-faciliated MAM formation is a key cause of CP-resistance and interruption of MAM integrity by GRP75 targeting has potential to overcome CP-resistance.

## Supplementary Material

Supplementary figures and tables.

## Figures and Tables

**Table 1:** Clinicopathological features of OC patients and association with expression of MAM components

Variables (all cases=72)	Cases	GRP75-high % (n)	VDAC1-high % (n)	IP3R-high % (n)
**Age (years)**				
<55	32	21 (65.63)	27 (84.38)	13 (40.63)
≥55	40	28 (70.00)	32 (80.00)	21 (52.50)
(X^2^) *P*		(0.03) 0.86	(0.02) 0.88	(0.36) 0.55
**Histological grade**				
Middle-high differentiation 3+4	30	11 (36.67)	19 (63.33)	17 (56.67)
Low differentiation 1+2	42	38 (90.48)	40 (95.24)	17 (40.48)
(X^2^) *P*		(4.81) 0.03	(1.24) 0.27	(0.65) 0.42
**Histological type**				
Serous cystadenocarcinoma	47	32 (68.09)	39 (82.98)	19 (40.43)
Mucinous cytadenocarcinoma	10	8 (80.00)	9 (90.00)	4 (40.00)
Endometrioid carcinoma	7	6 (85.71)	6 (85.71)	6 (85.71)
Others	8	3 (37.50)	5 (62.50)	5 (62.50)
(X^2^) *P*		(1.09) 0.78	(0.28) 0.96	(1.83) 0.61
**FIGO stage**				
I-II	43	20 (46.51)	32 (74.42)	18 (41.86)
III-IV	29	29 (100.00)	27 (93.10)	16 (55.17)
(X^2^) *P*		(4.18) 0.04	(0.40) 0.53	(0.43) 0.51
**TNM stage**				
I-II	41	18 (43.90)	29 (70.73)	20 (48.78)
III-IV	31	31 (100.00)	30 (96.77)	14 (45.16)
(X^2^) *P*		(4.77) 0.03	(0.79) 0.37	(0.03) 0.86
**Tumor size (cm)**				
≤10	33	32 (96.97)	31 (93.94)	18 (54.55)
>10	39	17 (43.59)	28 (71.79)	16 (41.03)
(X^2^) *P*		(4.45) 0.04	(0.58) 0.44	(0.47) 0.49
**Metastasis (oviduct, uterus, pelvic peritoneum and lymph nodes)**				
Negative	27	4 (14.81)	14 (51.85)	10 (37.04)
Positive	45	45 (100.00)	45 (100.00)	24 (53.33)
(X^2^) *P*		(13.17) 0.00	(2.86) 0.09	(0.66) 0.41
Tissue markers*				
Ki67 (-)	36	25 (69.44)	27 (75.00)	17 (47.22)
Ki67 (+)	23	22 (95.65)	22 (95.65)	12 (52.17)
(X^2^) *P*		(0.66) 0.42	(0.39) 0.53	(0.05) 0.83
MtP53 (-)	25	20 (80.00)	18 (72.00)	10 (40.00)
MtP53 (+)	34	27 (79.41)	31 (91.18)	19 (55.88)
(X^2^) *P*		(0.00) 0.99	(0.36) 0.55	(0.51) 0.48
GST-π (-)	22	10 (45.45)	12 (54.55)	7 (31.82)
GST-π (+)	37	37 (100.00)	37 (100.00)	22 (59.46)
(X^2^) *P*		(3.18) 0.07	(2.03) 0.15	(1.52) 0.22
P-gp (-)	21	12 (57.14)	18 (85.71)	10 (47.62)
P-gp (+)	38	35 (92.11)	31 (81.58)	19 (50.00)
(X^2^) *P*		(1.24) 0.27	(0.02) 0.90	(0.01) 0.92
CA125 (-)	13	9 (69.23)	9 (69.23)	10 (76.92)
CA125 (+)	59	40 (67.80)	50 (84.75)	24 (40.68)
(X^2^) *P*		(0.00) 0.97	(0.18) 0.67	(1.75) 0.19

* IHC Staining data of Ki67, P53, GST-π and P-gp were not available in 13 cases. Other IHC markers (TOPOⅡ, PCNA, PR, and ER) were not available during interval debulking surgery and not included here. *P* values represent the results of the Chi-square test.

## Abbreviations

ANT: adenine nucleotide translocase
ATP: adenosine triphosphate
CNX: calnexin
CP: cisplatin
co-IP: co-immunoprecipitation
CypD: cyclophilin D
[Ca^2+^]_I_: cytosolic Ca^2+^
[Ca^2+^]_m_: mitochondrial Ca^2+^
ΔΨm: mitochondrial membrane potential
EC: endometrioid adenocarcinoma
ER: endoplasmic reticulum
GRP75: 75-kDa glucose-regulated protein
HGSC: high-grade serous ovarian cancer
IHC: immunohistochemistry
iROS: cytosolic ROS
IP3R: inositol 1,4,5-trisphosphate receptor
KD: knock-down
MAM: mitochondria-associated ER membrane
Mc: crude mitochondria
MC: mucinous adenocarcinoma
MCC: co-localization coefficient
MCU: mitochondrial Ca^2+^ uniporter
MDR: multidrug resistance
Mito: mitochondria
mtROS: mitochondrial ROS
Mp: pure mitochondria
mPTP: mitochondrial permeability transition
mtDNA: mitochondrial DNA
NAD^+^: oxidized nicotinamide adenine dinucleotide
NADH: reduced nicotinamide adenine dinucleotide
NC: negative control
nDNA: nuclear DNA
NS: no significant difference
OC: ovarian cancer
OE: overexpression
OXPHOS: oxidative phosphorylation
PLA: proximity ligation assay
ROS: reactive oxygen species
SC: serous cystadenocarcinoma
TEM: transmission electron microscopy
TCA: tricarboxylicacid
VDAC1: voltage-dependent anion channel
