# Supplementary material for: GRP75-faciliated Mitochondria-associated ER Membrane (MAM) Integrity controls Cisplatin-resistance in Ovarian Cancer Patients
Source: Int J Biol Sci. 2022 Apr 11;18(7):2914–31. doi: 10.7150/ijbs.71571 (PMC9066115; doi:10.7150/ijbs.71571)
Supplement: Supplementary file 1 — Supplementary figures and tables. [file ijbsv18p2914s1.pdf]

Supplementary

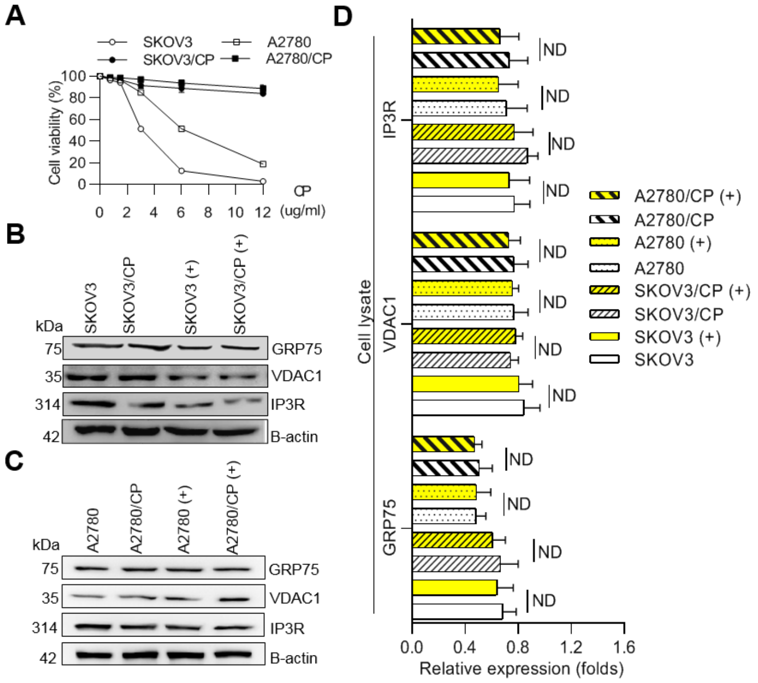

Fig. S1 Expression levels of MAM components in OC cells. (**A**) CP-sensitive and -resistant OC cells were exposed to CP at various concentrations. CCK-8 assays were used to determine the IC50 values of paired cell lines. (**B**, **C**) Western blotting of GRP75, VDAC1 and IP3R level in the total lysate of OC cells. (+) CP exposure: 4ug/mL for SKOV3 and SKOV3/CP cells, and 8ug/mL for A2780 and A2780/CP cells. (**D**) Expression quantification of MAM components in CP-sensitive and -resistant OC cells with/without CP exposure.

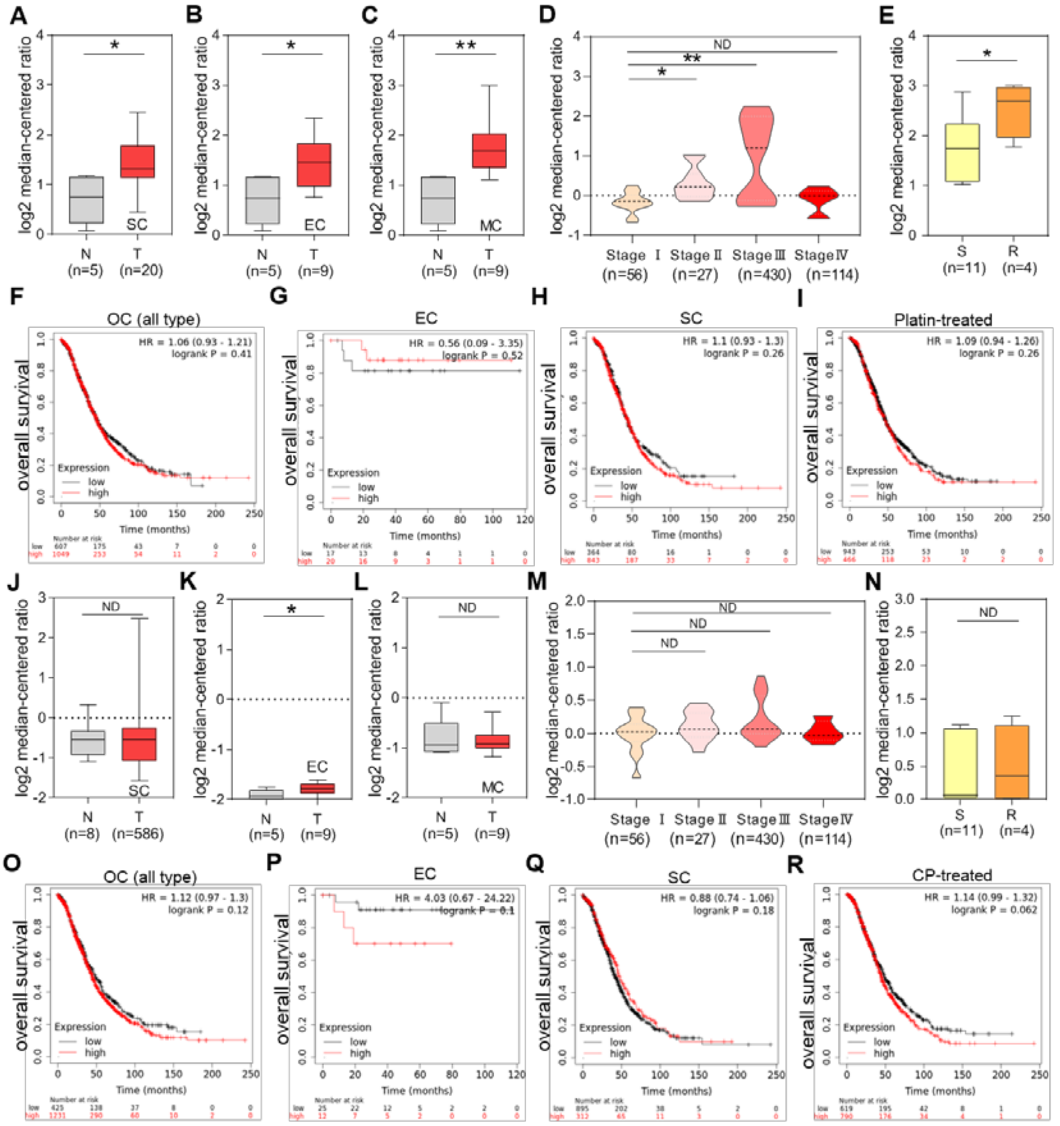

Fig. S2 Bioinformatics analysis of the correlation of VDAC1 and IP3R expression with OC patients' malignant phenotypes. Oncomine analysis of VDAC1 and IP3R mRNA expression in clinical samples. Box plots depict the expression level of VDAC1 and IP3R between normal (N) tissues and OC (T) with SC type (**A**, **J**), EC type (**B**, **K**), MC type (**C**, **L**), among different pathological stages (**D**, **M**), and between CP-sensitive (S) and -resistant (R) OC cell lines (**E**, **N**). Student's t-test, **P* < 0.05, ***P* < 0.01. Survival plots depict the OS for VDAC1 and IP3R expression-different patients with different OC types: All types (**F**, **O**), EA type (**G**, **P**), MA type (**H**, **Q**), and with CP treatment (**I**, **R**). Log-rank test. SC, Serous cystadenocarcinoma. MC, Mucinous cytadenocarcinoma. EC, Endometrioid carcinoma.

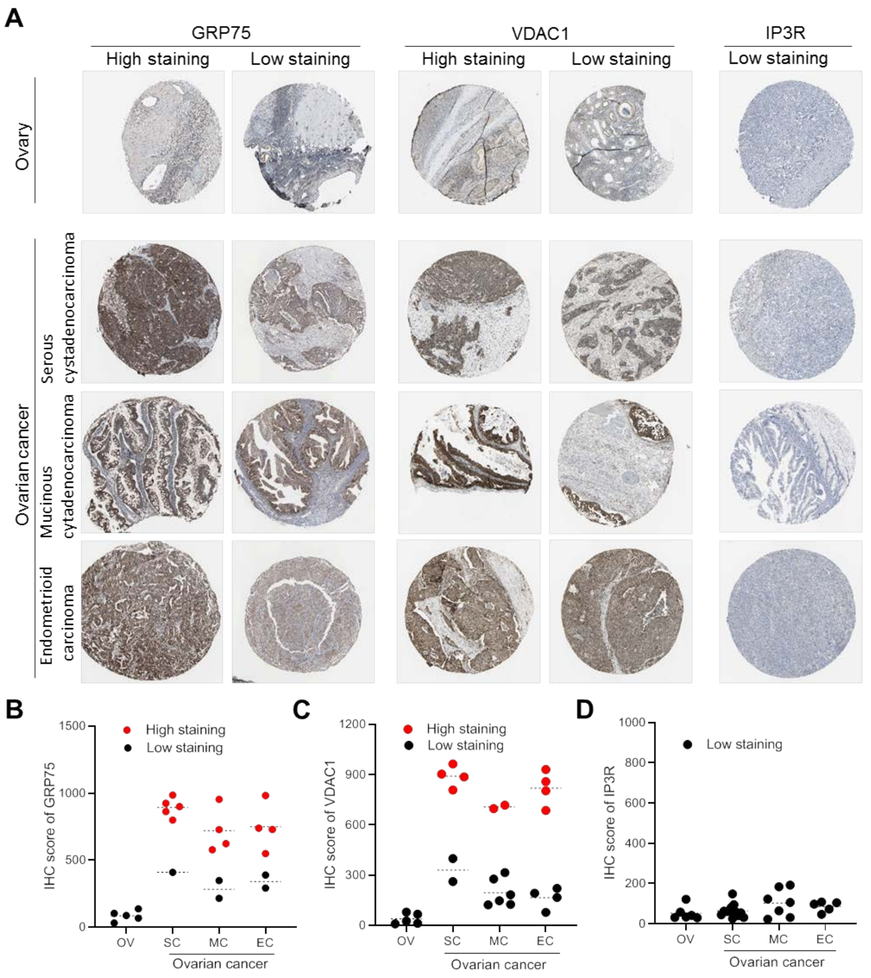

Fig. S3 Protein expression profiles of MAM components in OC tissues. (**A**) Representative IHC staining of GRP75, VDAC1, and IP3R in OC tissue microarrays obtained from the Human Protein Atlas. (**B**, **C**, **D**) IHC quantification of protein expression in OC samples with different histological types. OV, Ovary. SC, Serous cystadenocarcinoma. MC, Mucinous cytadenocarcinoma. EC, Endometrioid carcinoma.

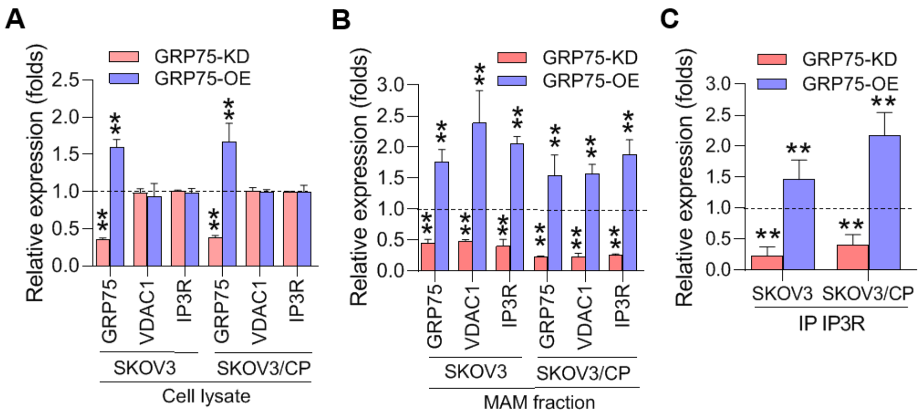

Fig. S4 GRP75-KD weakens the enrichment of other components of MAM in OC cells. (**A** and **B**)

Western blotting quantification of MAM components in CP-sensitive and -resistant OC cells after GRP75-KD or -OE. (**C**) Quantification of IP3R-immunoprecipited VDAC1 in GRP75-expression-changed, CP-sensitive, and –resistant OC cells. Data are mean ± SD based on 3 independent experiments, Student's t-test, $**P < 0.01$.

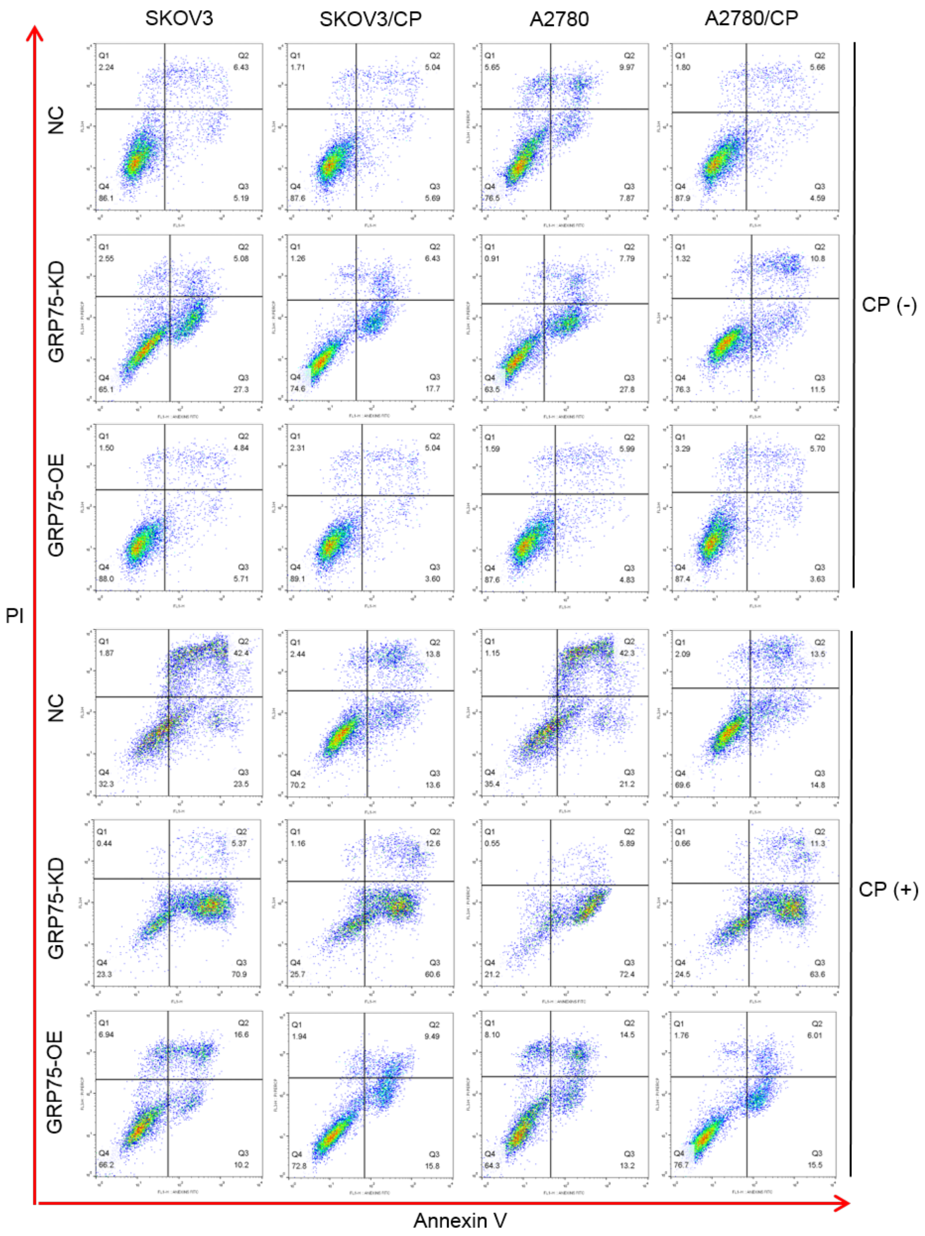

Fig. S5 Flow cytometry-based detection of apoptosis in GRP75-expression-changed OC cells with/without CP exposure. Negative cells for both fluorochromes (lower left) represents living target cells, annexin V^+ cells (lower right) characterize early-stage apoptotic cells, annexin V^+/PI^+ (upper right) cells identify late-stage apoptotic cells, and PI^+ (upper left) cells represent cells with a permeabilized membrane. Representative results are shown.

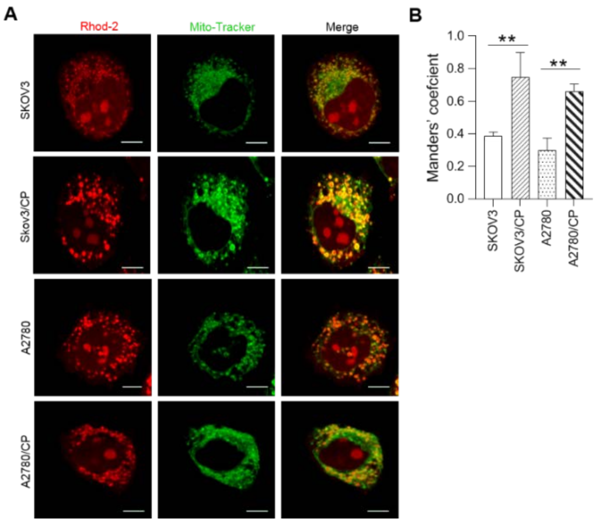

Fig. S6 High mitochondrial Ca^{2+} level was detected in CP-resistant OC cells. (**A**) Representative confocal imaging of the loaded Rhod-2 (Red) and Mito-Tracker (Green) signal in OC cells. (**B**) Quantification of $[Ca^{2+}]_m$ level in CP-sensitive and -resistant OC cells. Mean ± SD; n=60 cells per group. Student's t-test, $^{**}P < 0.01$.

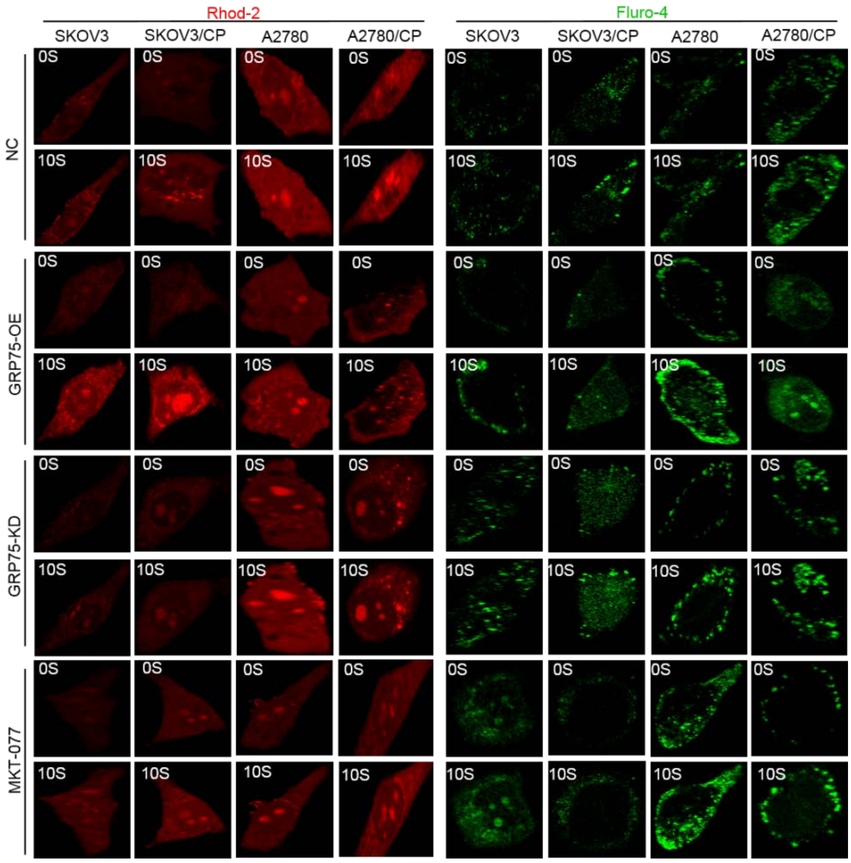

Fig. S7 Representative confocal imaging of mitochondrial and cytosolic Ca^{2+} in OC cells. Time-course mode-based confocal imaging of the changes of $[Ca^{2+}]_m$ (Rhod-2 signal) and $[Ca^{2+}]_i$ (Fluo-4 signal) levels in CP-treated OC cells after GRP75-KD, -OE, or inhibition with MKT-077.

Table S1 Primary antibodies used in this study.

Assays	Primary antibody	Dilution
PLA		
	Mouse anti-VDAC1	1:100
	Rabbit anti-IP3R	1:500
Western Blot		
	Rabbit anti-CNX	1:2000
	Mouse anti-GRP75	1:1000
	Rabbit anti-Cyto C	1:2000
	Mouse anti-VDAC1	1:1000
	Rabbit anti-IP3R	1:1000
	Mouse anti-Tubulin	1:1000
	Mouse anti-β-actin	1:20000
	Rabbit anti-Caspase 3	1:500
	Rabbit anti-Caspase 9	1:1000
	Rabbit anti-Calpain 1	1:800
Co-IP		
	Mouse anti-VDAC1	1:100
IHC		
	Mouse anti-GRP75	1:100
	Rabbit anti-IP3R	1:100
	Rabbit anti-VDAC1	1:30

Table S2 Clinicopathological features of OC patients and association with the co-expression of GRP75, VDAC1, and IP3R.

Variables (all cases=72)	GRP75+VDAC1 high %(n)	GRP75+IP3R high %(n)	VDAC1+IP3R high %(n)	GRP75+VDAC1+IP3R high %(n)
Age (years)				
<55 (32)	17 (53.13)	10 (31.25)	13(40.63)	8 (25.00)
≥55 (40)	26 (65.00)	18 (45.00)	12 (30.00)	12 (30.00)
(X^2) *P*	(0.27) 0.61	(0.63) 0.43	(0.43) 0.51	(0.13) 0.72
Histological grade				
Middle-high differentiation 3+4 (30)	9 (30.00)	11(36.67)	10 (33.33)	6 (20.00)
Low differentiation 1+2 (42)	34 (80.95)	17 (40.48)	15 (35.71)	14 (33.33)
(X^2) *P*	(5.17) 0.02	(0.05) 0.83	(0.02) 0.88	(0.89) 0.34
Histological type				
Serous cystadenocarcinoma (47)	32 (68.09)	16 (34.04)	19 (40.43)	15 (31.91)
Mucinous cytadenocarcinoma (10)	7 (70.00)	4 (40.00)	2 (20.00)	2 (20.00)
Endometrioid carcinoma (7)	4 (57.14)	5 (71.43)	3 (42.86)	3 (42.86)

Others (8)	0 (0.00)	3 (37.50)	1 (12.50)	0 (0.00)
(X^2) *P*	(5.21) 0.16	(1.33) 0.72	(1.94) 0.59	(3.02) 0.39
FIGO stage				
Ⅰ-Ⅱ(43)	16 (37.21)	12 (27.91)	9 (20.93)	5 (11.63)
Ⅲ-Ⅳ(29)	27 (93.10)	16 (55.17)	16 (55.17)	15 (51.72)
(X^2) *P*	(5.46) 0.02	(2.32) 0.13	(4.20) 0.04	(7.56) 0.01
TNM stage				
Ⅰ-Ⅱ(41)	15 (36.59)	14 (34.15)	11 (26.83)	6 (14.63)
Ⅲ-Ⅳ(31)	28 (90.32)	14 (45.16)	14 (45.16)	14 (45.16)
(X^2) *P*	(5.24) 0.02	(0.39) 0.53	(1.25) 0.26	(4.55) 0.03
Tumor size (cm)				
≤10 (33)	30 (90.91)	18 (54.55)	16 (48.48)	16 (48.48)
＞10 (39)	13 (33.33)	10 (25.64)	9 (23.08)	4 (10.26)
(X^2) *P*	(6.23) 0.01	(2.75) 0.10	(2.45) 0.12	(7.34) 0.01
Metastasis (oviduct, uterus, pelvic peritoneum and lymph nodes)				
Negative (27)	3 (11.11)	4 (14.81)	4 (14.81)	2 (7.41)
Positive (45)	40 (88.89)	24 (53.33)	21 (46.67)	18 (40.00)
(X^2) *P*	(5.19) 0.03	(5.08) 0.02	(3.95) 0.05	(5.48) 0.02
Tissue markers *				
Ki67 (-) (36)	18 (50.00)	8 (22.22)	11 (30.56)	8 (22.22)
Ki67 (+) (23)	22 (95.65)	12 (52.17)	11 (47.83)	10 (43.48)
(X^2) *P*	(2.47) 0.12	(2.67) 0.10	(0.80) 0.37	(1.55) 0.21
MtP53 (-) (25)	15 (60.00)	9 (36.00)	10 (40.00)	7 (28.00)
MtP53 (+) (34)	25 (73.53)	11 (32.35)	12 (35.29)	11 (32.35)
(X^2) *P*	(0.24) 0.63	(0.04) 0.84	(0.06) 0.80	(0.07) 0.79
GST-π (-) (22)	5 (22.73)	2 (9.09)	6 (27.27)	2 (9.09)
GST-π (+) (37)	35 (94.59)	18 (48.65)	16 (43.24)	16 (43.24)
(X^2) *P*	(7.39) 0.01	(5.26) 0.02	(0.71) 0.40	(4.41) 0.04
P-gp (-) (21)	11 (52.38)	3 (14.29)	7 (33.33)	3 (14.29)
P-gp (+) (38)	29 (76.32)	17 (44.74)	15 (39.47)	15 (39.47)
(X^2) *P*	(0.71) 0.40	(3.00) 0.08	(0.10) 0.75	(2.30) 0.13
CA125 (-) (13)	4 (30.77)	9 (69.23)	9 (69.23)	4 (30.77)
CA125 (+) (59)	39 (66.10)	19 (32.20)	16 (27.12)	16 (27.12)
(X^2) *P*	(1.64) 0.20	(2.33) 0.13	(3.41) 0.06	(0.04) 0.84

* IHC Staining data of Ki67, P53, GST-π, and P-gp were not available in 13 cases.

P values represent the results of the Chi-square test.
